# Functional analysis of multiple *nifB* genes of *Paenibacillus* strains in synthesis of Mo-, Fe- and V-nitrogenases

**DOI:** 10.1186/s12934-021-01629-9

**Published:** 2021-07-19

**Authors:** Qin Li, Haowei Zhang, Liqun Zhang, Sanfeng Chen

**Affiliations:** 1 State Key Laboratory for Agrobiotechnology, College of Biological Sciences, China Agricultural University, Beijing, 100193 People’s Republic of China; 2 Key Laboratory of Pest Monitoring and Green Management, Ministry of Agriculture and Rural Affairs, and College of Plant Protection, China Agricultural University, Beijing, 100193 People’s Republic of China

**Keywords:** *Paenibacillus*, *nifB* gene, Mo-nitrogenase, Alternative nitrogenases

## Abstract

**Background:**

Biological nitrogen fixation is catalyzed by Mo-, V- and Fe-nitrogenases that are encoded by *nif*, *vnf* and *anf* genes, respectively. NifB is the key protein in synthesis of the cofactors of all nitrogenases. Most diazotrophic *Paenibacillus* strains have only one *nifB* gene located in a compact *nif* gene cluster (*nifBHDKENX*(*orf1*)*hesAnifV*). But some *Paenibacillus* strains have multiple *nifB* genes and their functions are not known.

**Results:**

A total of 138 *nifB* genes are found in the 116 diazotrophic *Paenibacillus* strains. Phylogeny analysis shows that these *nifB* genes fall into 4 classes: *nifB*I class including the genes (named as *nifB1* genes) that are the first gene within the compact *nif* gene cluster, *nifB*II class including the genes (named as *nifB2* genes) that are adjacent to *anf* or *vnf* genes, *nifB*III class whose members are designated as *nifB3* genes and *nifB*IV class whose members are named as *nifB4* genes are scattered on genomes. Functional analysis by complementation of the ∆*nifB* mutant of *P. polymyxa* which has only one *nifB* gene has shown that both *nifB1* and *nifB2* are active in synthesis of Mo-nitrogenase, while *nifB3* and *nifB4* genes are not. Deletion analysis also has revealed that *nifB1* of *Paenibacillus sabinae* T27 is involved in synthesis of Mo-nitrogenase, while *nifB3* and *nifB4* genes are not. Complementation of the *P. polymyxa* ∆*nifBHDK* mutant with the four reconstituted operons: *nifB1anfHDGK*, *nifB2anfHDGK*, *nifB1vnfHDGK* and *nifB2vnfHDGK,* has shown both that *nifB1* and *nifB2* were able to support synthesis of Fe- or V-nitrogenases. Transcriptional results obtained in the original *Paenibacillus* strains are consistent with the complementation results.

**Conclusions:**

The multiple *nifB* genes of the diazotrophic *Paenibacillus* strains are divided into 4 classes. The *nifB1* located in a compact *nif* gene cluster (*nifBHDKENX*(*orf1*)*hesAnifV*) and the *nifB2* genes being adjacent to *nif* or *anf* or *vnf* genes are active in synthesis of Mo-, Fe and V-nitrogenases, but *nifB3* and *nifB4* are not. The reconstituted *anf* system comprising 8 genes (*nifBanfHDGK* and *nifXhesAnifV*) and *vnf* system comprising 10 genes (*nifBvnfHDGKEN* and *nifXhesAnifV*) support synthesis of Fe-nitrogenase and V-nitrogenase in *Paenibacillus* background, respectively.

**Supplementary Information:**

The online version contains supplementary material available at 10.1186/s12934-021-01629-9.

## Background

Biological nitrogen fixation, a process unique to some bacteria and archaea (called diazotrophs), is catalyzed by nitrogenase and plays an important role in world agriculture [1]. There are three known nitrogenase designated as the Mo-nitrogenase, V-nitrogenase and Fe-nitrogenase that are encoded by *nif*, *vnf*, and *anf*, respectively [2]. Nitrogen fixation is mainly catalyzed by Mo-nitrogenase, which is found in all diazotrophs. In addition to Mo-nitrogenase, some possess either of alternative Fe-nitrogenase and V-nitrogenase, or both. Each nitrogenase contains two components, a catalytic protein and a reductase [3–5]. For Mo-nitrogenase, MoFe protein is the catalytic protein and Fe protein is the reductase. The MoFe protein is an α_2_β_2_ heterotetramer (encoded by *nifD* and *nifK*) that contains two metal clusters: FeMo-co, a [Mo-7Fe-9S-C-homocitrate] cluster which serves as the active site of N_2_ binding and reduction and the P-cluster, a [8Fe-7S] cluster which shuttles electrons to FeMo-co. The Fe protein (encoded by *nifH*) is a homodimer bridged by an intersubunit [4Fe-4S] cluster that serves as the obligate electron donor to the MoFe protein [6–8]. Like Mo-nitrogenase, alternative nitrogenases comprise an electron-delivery Fe protein (encoded by *anfH* in Fe-nitrogenase and encoded by *vnfH* in V-nitrogenase). The FeFe protein of Fe-nitrogenase encoded by *anfDK* and the VFe protein of V-nitrogenase encoded by *vnfDK* are homologous to the MoFe protein of Mo-nitrogenase. The alternative nitrogenases have either FeFe-co or FeV-co at the active site and also include an additional subunit (AnfG or VnfG) encoded by *anfG* or *vnfG* [9]. The FeFe-co is analogous to FeMo-co except for containing Fe in place of Mo [10], but FeV-co is a [V–7Fe–8S–C-homocitrate] cluster which replaces Mo with V and lacks one S compared to FeMo-co [11].

NifB has been demonstrated to be essential for the synthesis of all nitrogenases. NifB is a radical S-adenosyl methionine (SAM) enzyme that catalyzes the formation of NifB-co, a [8Fe-9S-C] cluster which is a common precursor for the syntheses of FeMo-co of Mo-nitrogenase, FeV-co of V-nitrogenase and FeFe-co of Fe-nitrogenase [12–14]. NifB-co is subsequently transferred to the scaffold protein NifEN, upon which mature cofactor is synthesized. The NifX protein is known to bind NifB-co and involved in NifB-co transfer [15].

The number, structure and properties of *nifB* genes show some variation among different diazotrophs. *Azotobacter vinelandii* and *Rhodopseudomonas palustris* possess only one *nifB* gene that is responsible for three types of nitrogenases and mutation of *nifB* gene led to loss of all nitrogenases activities [16, 17]. *Rhodobacter capsulatus* with Mo-nitrogenase and Fe-nitrogenase carries two *nifB* genes that are located in two *nif* gene clusters [18] and either one of the two *nifB* genes was sufficient for nitrogen fixation via the Mo-dependent or Fe-dependent nitrogenase [19]. The cyanobacterium *Anabaena variabilis* ATCC 29,413 has two *nifB* genes for synthesis of two Mo-nitrogenases, but *nifB1* is specifically expressed in heterocysts and *nifB2* is specifically expressed in vegetative cells [20]. On the basis of NifB domain architecture, the NifB proteins are divided into three subfamilies [21, 22]. The first NifB subfamily has an N-terminal SAM-radical domain linked to a C-terminal NifX-like domain. A major of NifB proteins from Bacteria domain (e.g. *A. vinelandii* and *Klebsiella oxytoca*) belong to the first NifB subfamily. The second NifB subfamily contains a stand-alone SAM-radical domain and is found in Bacteria and Archaea domains. The third NifB subfamily has three domains including a NifN-like domain, a SAM-radical domain and a C-terminal NifX-like domain and is found in *Clostridium* species.

The *Paenibacillus* genus of the Firmicutes phylum is a large one that currently comprises 254 validly named species (https://www.bacterio.net/paenibacillus.html), more than 20 of which have the nitrogen fixation ability [23]. Comparative genome sequence analysis of 15 diazotrophic *Paenibacullus* strains have revealed that a compact *nif* gene cluster comprising 9–10 genes (*nifB nifH nifD nifK nifE nifN nifX* (*orf1*) *hesA nifV*) encoding Mo-nitrogenase is conserved in the N_2_-fixing *Paenibacillus* genus [24]. The 9 genes (*nifBHDKENXhesAnifV*) in *Paenibacillus polymyxa* WLY78 are organized as an operon under control of a σ^70^ dependent promoter located in front of *nifB* gene [25]. In addition to the *nif* gene cluster, additional *nif* genes or *anf* or *vnf* genes are found in some diazotrophic *Paenibacillus* spp. For examples, *Paenibacillus sabinae* T27 has multiple *nifB*, *nifH*, *nifE* and *nifN* genes [26]. *Paenibacillus forsythia* T98 and *Paenibacillus sophorae* S27 have additional *nif* and *anfDHGK* genes, *Paenibacillus zanthoxyli* JH29 and *Paenibacillus durus* (previously called as *Paenibacillus azotofixans*) ATCC 35681 contain additional *nif* and *vnfDHGKEN* genes [24]. Notably, in addition to the *nifB* gene in the compact *nif* gene cluster comprising 9–10 genes (*nifB nifH nifD nifK nifE nifN nifX* (*orf1*) *hesA nifV*) encoding Mo-nitrogenase, multiple *nifB* genes are found in some *Paenibacillus* species that carry additional *nif* genes or *anf* genes or *vnf* genes [24, 26]. However, functions of the multiple *nifB* genes are not known. In this study, we analyzed the distribution and phylogeny of the 138 putative NifB proteins from 116 diazotrophic *Paenibacillus* strains. All *nifB* genes in *Paenibacillus* fall into 4 classes: *nifB*I, *nifB*II, *nifB*III and *nifB*IV. We demonstrate that only *nifB*I and *nifB*II are functional in synthesis of Mo-, Fe- and V-nitrogenase. The *nifB*III and *nifB*IV may be not involved in nitrogen fixation. In addition, the reconstituted *anf* system comprising 8 genes (*nifBanfHDGK* and *nifXhesAnifV*) and *vnf* system comprising 10 genes (*nifBvnfHDGKEN* and *nifXhesAnifV*) supported synthesis of Fe-nitrogenase and V-nitrogenase in *Paenibacillus* background, respectively. Our study will provide guidance for engineering nitrogenase into heterologous hosts.

## Results

### Classification of *nifB* genes of *Paenibacillus* genus

Here, the nitrogen fixation genes in the genomes of the 116 diazotrophic *Paenibacillus* strains taken from the RefSeq database are comparatively analyzed (Additional file 1: Table S1). A compact *nif* gene cluster composed of 9–10 genes (*nifBHDKENX*(*orf1*)*hesAnifV*) is conserved in all of the diazotrophic strains, in agreement with the previous studies [24]. In addition to the compact *nif* gene cluster encoding Mo-nitrogenase, 9 strains have additional *anfHDGK* encoding Fe-nitrogenase and 3 strains have additional *vnfHDGKEN* encoding V-nitrogenase.

A total of 138 NifB putative sequences are found in the 116 diazotrophic *Paenibacillus* strains. According to the *nifB* sequence similarity, the *nifB* genes were divided into 4 classes. The *nifB*I class includes the *nifB* genes (named as *nifB1* genes) that are the first gene in the compact *nif* gene cluster comprising 9–10 genes (*nifB nifH nifD nifK nifE nifN nifX* (*orf1*) *hesA nifV*) and the genes linked to another *nifH.* The *nifB*II class includes these genes (named as *nifB2* genes) that are linked to additional copies of *nifENXorf*(*fer*) genes preceding *anfHDGK* or additional copies of *nifENXorforf* genes preceding *vnfHDGKEN* or *orforf* preceding *vnfHDGKEN.* The genes (named as *nifB3*) of *nifB*III class and the genes (named as *nifB4*) of *nifB*IV are scattered at different locations of genomes.

**Fig. 1:**
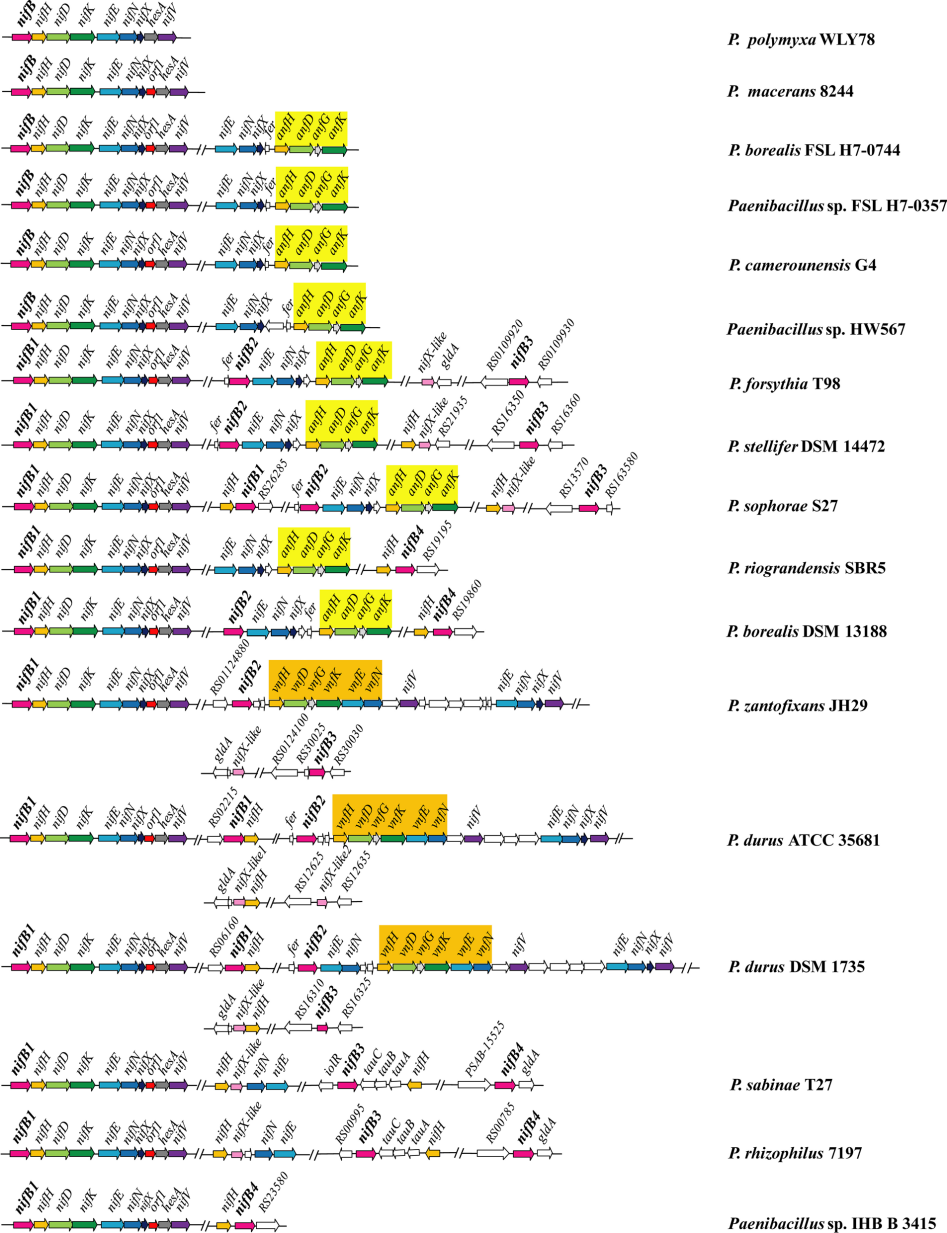
Genetic organization of the *nifB* loci and other *nif*, *anf*, *vnf* genes in N_2_-fixing *Paenibacillus* strains. The compact *nif* gene cluster comprising contiguous 9–10 genes *nifBHDKENX*(*orf1*)*hesAnifV*. The *anf* genes are marked with yellow color and the *vnf* genes are marked with apricot yellow. The *nifB* genes are shown in magenta. The *nifX*-like genes whose predicted products show high sequence similarity with the C-terminal domain of NifB are shown in pink

Of the 116 diazotrophic *Paenibacillus* strains, 105 strains have only one *nifB* and 11 strains have 2–4 *nifB* genes. *Paenibacillus polymyxa* WLY78 is a representative that has only a *nifB1* located in the compact *nif* gene cluster consisting of 9 genes (*nifBHDKENXhesAnifV*) encoding Mo-nitrogenase (Fig. 1 and Additional file 1: Table S1). *Paenibacillus sabinae* T27 is a representative strain with three *nifB* genes (*nifB1*, *nifB3* and *nifB4*), but contained only Mo-nitrogenase. For the strains with both Mo- and V-nitrogenases, *Paenibacillus zanthoxyli* JH29 has *nifB1*, *nifB2* and *nifB3*, but *Paenibacillus durus* DSM 1735 has *nifB2*, *nifB3* and 2 copies of *nifB1*: one being located in the compact *nif* cluster and the other being linked to another *nifH.* For the strains with both Mo- and Fe-nitrogenases, *Paenibacillus forsythiae* T98 has three *nifB* genes (*nifB1*, *nifB2* and *nifB3*), whereas *Paenibacillus sophorae* S27 has four *nifB* genes (*nifB2*, *nifB3*, and 2 copies of *nifB1*). The other 4 strains (*Paenibacillus borealis* FSL H70744, *Paenibacillus* sp. FSL H7-0357, *Paenibacillus* sp. HW567 and *Paenibacillus camerounensis* G4) with both Mo- and Fe-nitrogenases possess only one *nifB* gene. Organization of the *nifB* genes and other nitrogen fixation genes from 17 representatives of *Paenibacillus* strains is shown in Fig. 1.

### Phylogeny and structure of *Paenibacillus* NifB proteins

**Fig. 2:**
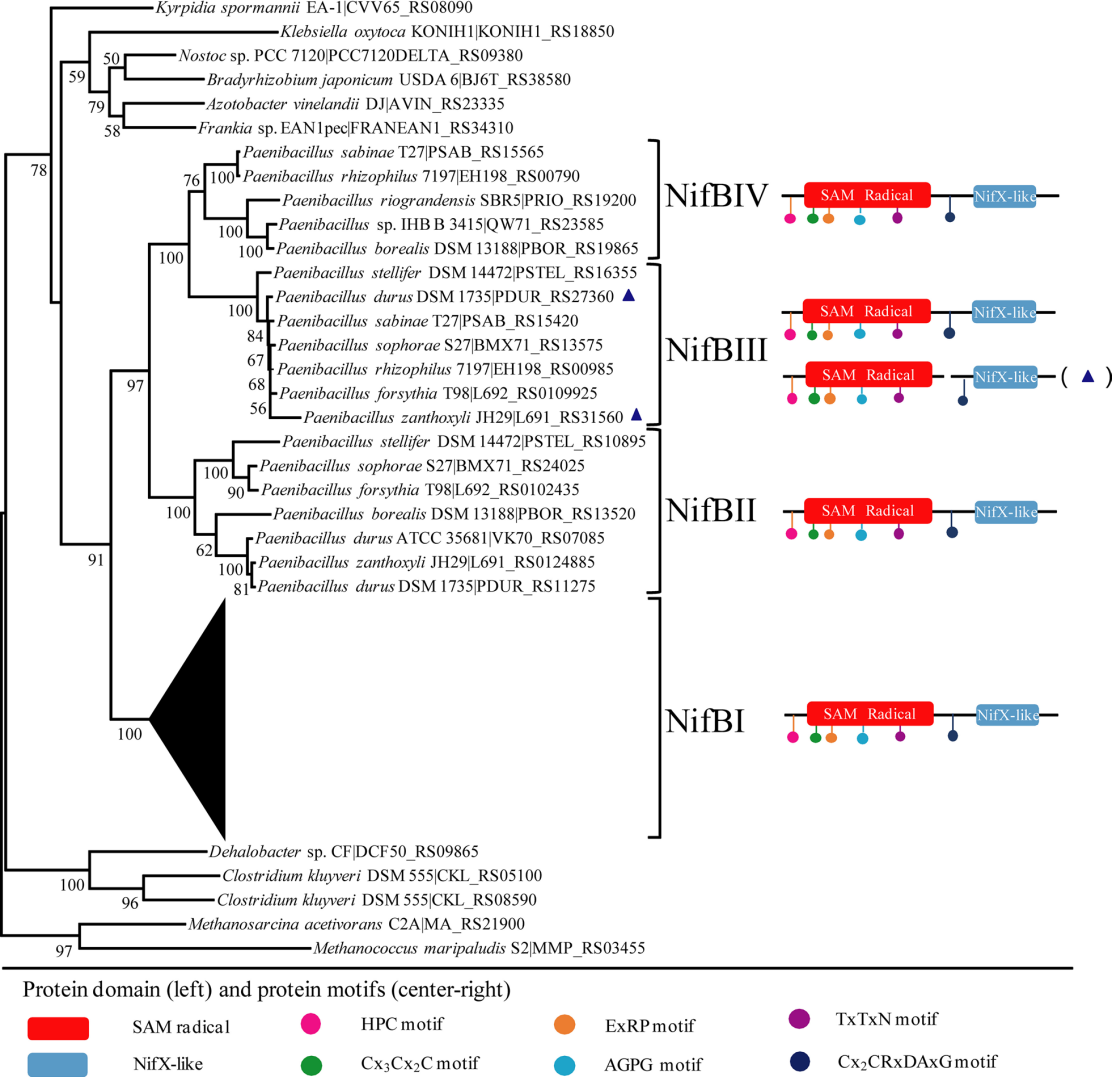
Maximum likelihood phylogenetic tree and architectures of NifB proteins from N_2_-fixing *Paenibacillus* strains. All the NifB1 proteins in N_2_-fixing *Paenibacillus* strains clustered together and were not shown. The SAM-radical is shown in red and the NifX-like domain in blue. Color dots represent conserved motifs in the NifB proteins. The NifB has only a stand-alone SAM-radical domain marked blue triangle

Here, 138 putative NifB sequences from 116 diazotrophic *Paenibacillus* strains are used to construct a phylogenetic tree, with 11 NifB sequences from 10 diazotrophs (*A. vinelandii*, *K. oxytoca*, *Bradyrhizobium japonicum*, *Clostridium kluyveri, Dehalobacter* sp*., Kyrpidia spormannii*, *Methanosarcina acetivorans*, *Methanococcus maripaludis*, *Frankia* sp. EAN1pec, *Nostoc* sp. PCC 7120) as control (Fig. 2 and Additional file 1: Table S1). The phylogenetic tree has shown that all *Paenibacillus* putative NifB proteins form a large class which is separated from the NifB proteins from other diazotrophs. The data suggest that all *Paenibacillus* putative *nifB* genes have a common ancestor. The *Paenibacillus* putative NifB proteins are divided into 4 classes: NifBI, NifBII, NifBIII and NifBIV, which corresponded to the 4 *nifB* classes that are classified on basis of *nifB* sequence similarities. The NifB1, NifB2, NifB3 and NifB4 proteins corresponded to NifBI, NifBII, NifBIII and NifBIV classes, respectively. Phylogeny analyses have shown that the NifB1 proteins are emerged firstly in the diazotrophic *Paenibacillus* species, and NifB2, NifB3 and NifB4 may result from gene duplication.

Protein structure analysis showed that *Paenibacillus* NifB1, NifB2 and NifB4 have the same structure composed of an N-terminal SAM-radical domain and a C-terminal NifX-like domain. Most NifBIII members possesses the two domains, but the NifB3 proteins from the 2 strains (*P. zanthoxyli* JH29 and *P. durus* DSM 1735) have only a SAM-radical domain. The *Paenibacillus* NifB1, NifB2, NifB3 and NifB4 proteins that possess both domains are composed of 427–505 amino acids (Additional file 1: Table S1) and have similarity (> 57%) at amino acid levels. These proteins have a number of conserved motifs in the SAM-radical domain, including HPC motif, Cx_3_Cx_2_C motif, ExRP motif, AGPG motif, TxTxN motif and Cx_2_CRxDAxG (Fig. 2). However, the NifB3 proteins of *P. zanthoxyli* JH29 and *P. durus* DSM 1735 have only a SAM-radical domain that lacks the Cx2CRxDAxG motif. Sequence alignment of 13 NifB proteins including NifB1, NifB2, NifB3 and NifB4 from 4 representatives of *Paenibacillus* strains (*P. polymyxa* WLY78, *P. sabinae* T27, *P. forsythia* T98 and *P. zanthoxyli* JH29) is shown in Additional file 1: Figure S1.

### Transcription analysis of multiple *nifB* genes in medium containing only Mo or Fe or V

**Fig. 3:**
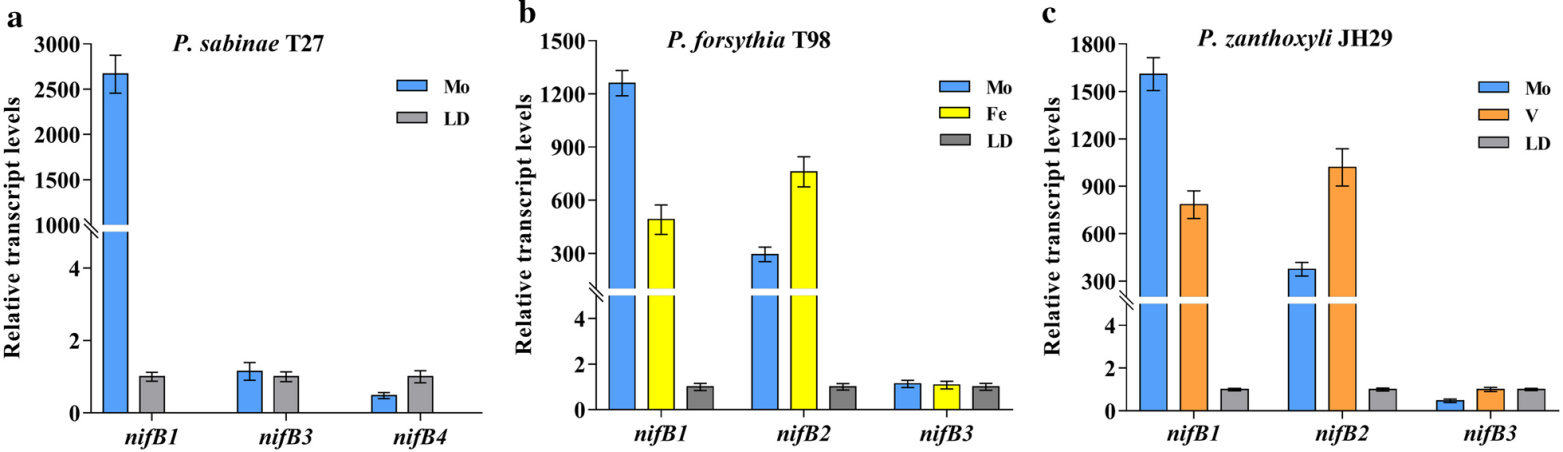
Transcription profile of the multiple *nifB* genes from *P. sabinae* T27(**a**), *P. forsythia* T98(**b**) and *P. zanthoxyli* JH29(**c**). RT-qPCR analysis of the relative transcript levels of the *nifB* genes in these *Paenibacillus* species grown in Mo-dependent, Fe-dependent and V-dependent nitrogen fixation conditions, with non-nitrogen fixing conditions of N-rich (LD medium) cultures as negative controls. The data are the mean of three biological replicates

As described above, *P. sabinae* T27 with only Mo-nitrogenase has NifB1, NifB3 and NifB4, *P. zanthoxyli* JH29 with both Mo- and V-nitrogenases has NifB1, NifB2 and NifB3 and *P. forsythiae* T98 with both Mo- and Fe-nitrogenases possesses NifB1, NifB2 and NifB3. The three species *P. sabinae* T27, *P. forsythia* T98 and *P. zanthoxyli* JH29 were used to investigate the transcriptions of the multiple *nifB* genes under different conditions by RT-qPCR. *Paenibacilllus sabinae* T27 was cultivated in Mo-dependent N_2_-fixing condition, while *P. forsythia* T98 and *P. zanthoxyli* JH29 were cultivated in Mo-dependent and Fe-dependent or V-dependent N_2_-fixing condition, respectively, with non-N_2_-fixing condition of N-rich (LD medium) cultures as negative controls (Fig. 3). For *P. sabinae* T27, the transcription level of *nifB1* exhibited more than 2000-fold of increase under Mo-dependent N_2_-fixing condition compared to under non-N_2_-fixing condition, but the transcripts from *nifB3* and *nifB4* showed no differences under both conditions (Fig. 3a). For *P. forsythia* T98 grown under both Mo-dependent and Fe-dependent condition, both *nifB1* and *nifB2* genes were highly transcribed, but *nifB3* was not induced by N_2_-fixing condition. The transcript level of *nifB1* was much higher in Mo-dependent condition than in Fe-dependent condition, while the transcript level of *nifB2* was higher in Fe-dependent condition than in Mo-dependent condition (Fig. 3b). For *P. zanthoxyli* JH29 grown under both Mo-dependent and V-dependent conditions, the transcription of both *nifB1* and *nifB2* genes were activated, but *nifB3* showed no differences in its expression under test conditions. The transcript level of *nifB1* was higher in Mo-dependent condition than in V-dependent condition, while the transcript level of *nifB2* was higher in V-dependent condition than in Mo-dependent condition (Fig. 3c). These results indicate that the *nifB1* and *nifB2* may be selectively expressed according to metal availability.

### Functional analysis of multiple *nifB* genes in synthesis of Mo-nitrogenase

**Fig. 4:**
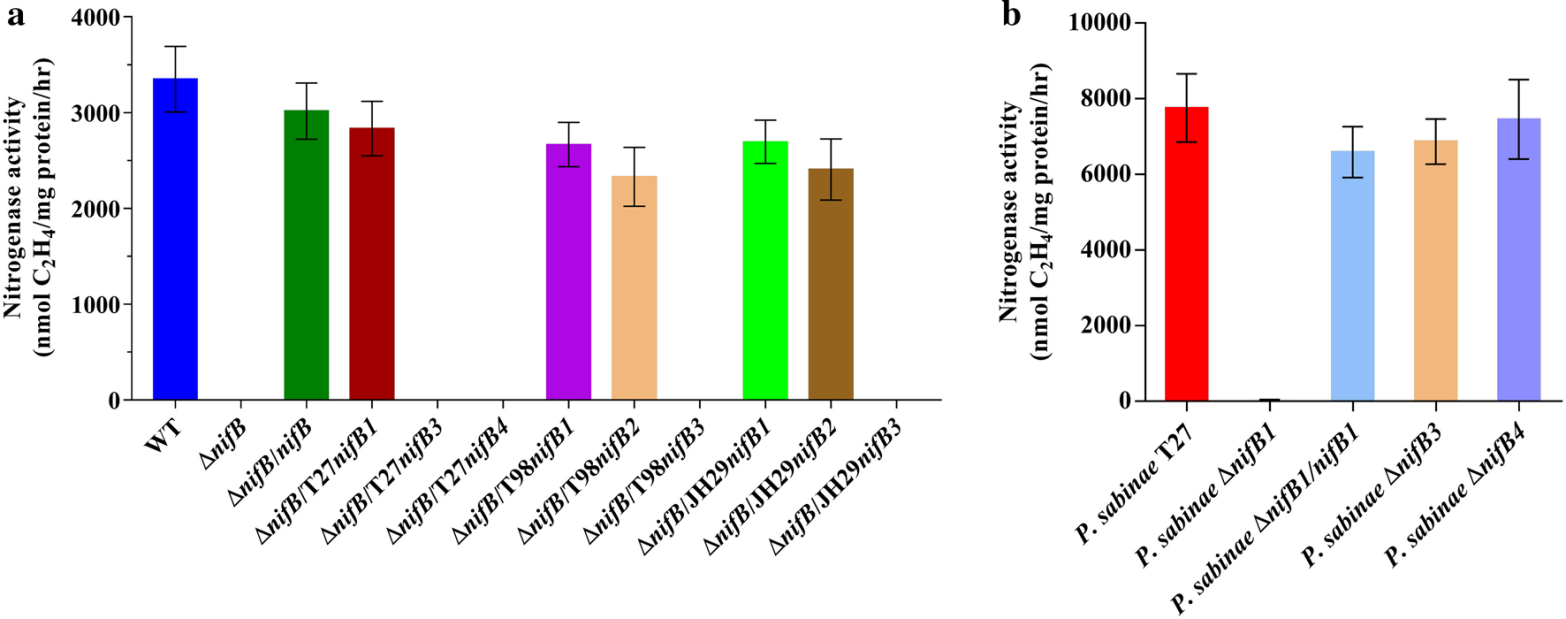
The nitrogenase activities of the *nifB* deletion mutants and their complementary strains in Mo-dependent nitrogen fixation conditions. **a** Nitrogenase activities of the *P. polymyxa* Δ*nifB* mutant and its complementary strains. **b** Nitrogenase activities of WT (*P. sabinae*T27), deletion mutants Δ*nifB1*, Δ*nifB3*, Δ*nifB4* and complementary strain Δ*nifB1/nifB1*. The nitrogenase activity was measured by acetylene reduction assay when bacterial cells were grown anaerobically in nitrogen limited medium containing Mo. Error bars indicate the SD observed from at least three independent experiments

The *nifB* deletion mutant (∆*nifB*) of *P. polymyxa* WLY78 was here constructed by using recombination method as described in materials and methods. The *P. polymyxa* ∆*nifB* mutant completely lost its nitrogenase activity and complementation by its *nifB* gene carried in a plasmid restored the nitrogenase activity (Fig. 4a). Thus, *P. polymyxa* ∆*nifB* mutant was used as a host for complementation to investigate the functionality of the multiple *nifB* genes. Each *nifB* gene from *P. sabinae* T27, *P. forsythia* T98 and *P. zanthoxyli* JH29 was cloned into a low-copy plasmid pRN5101[27, 28], in which the expression of these *nifB* genes were driven under the control of the *nifB* promoter of *P. polymyxa* (details are provided in materials and methods). Among the 3 *nifB* genes of *P. sabinae* T27, only the *nifB1* can effectively restore the nitrogenase activity of the *P. polymyxa* ∆*nifB* mutant, showing that the *nifB1* gene was transcribed under nitrogen fixation condition and the translated NifB1 was functional. Both *nifB1* and *nifB2* from *P. forsythia* T98 or *P. zanthoxyli* JH29 can effectively restore nitrogenase activity of the *P. polymyxa* ∆*nifB* mutant, but the *nifB3* from *P. forsythia* T98 or *P. zanthoxyli* JH29 can not restore activity. The result suggests that both *nifB1* and *nifB2* are functional in synthesis of Mo-nitrogenase, but *nifB3* product was not active.

To further examine the role of the multiple *nifB* genes, attempts to inactivate the *nifB* genes were made. Three single deletion mutants Δ*nifB1*, Δ*nifB3* and Δ*nifB4* of *P. sabinae* T27 were successfully constructed. Deletion of *nifB1* resulted to complete loss of nitrogenase activity. Whereas, the nitrogenase activities of Δ*nifB3* or Δ*nifB4* mutants were similar as that in wild-type *P. sabinae* T27 (Fig. 4b). The data are consistent with the above described qRT-PCR and heterologous complementation results, confirming that both *nifB3* and *nifB4* are not involved in nitrogen fixation. However, attempts to inactivate the *nifB* genes of *P. forsythia* T98 and *P. zanthoxyli* JH29 were not successful, due to hardness of genetic transformation in these strains.

### Functional analysis of *nifB1* and *nifB2* genes in synthesis of Fe- and V-nitrogenases

**Fig. 5:**
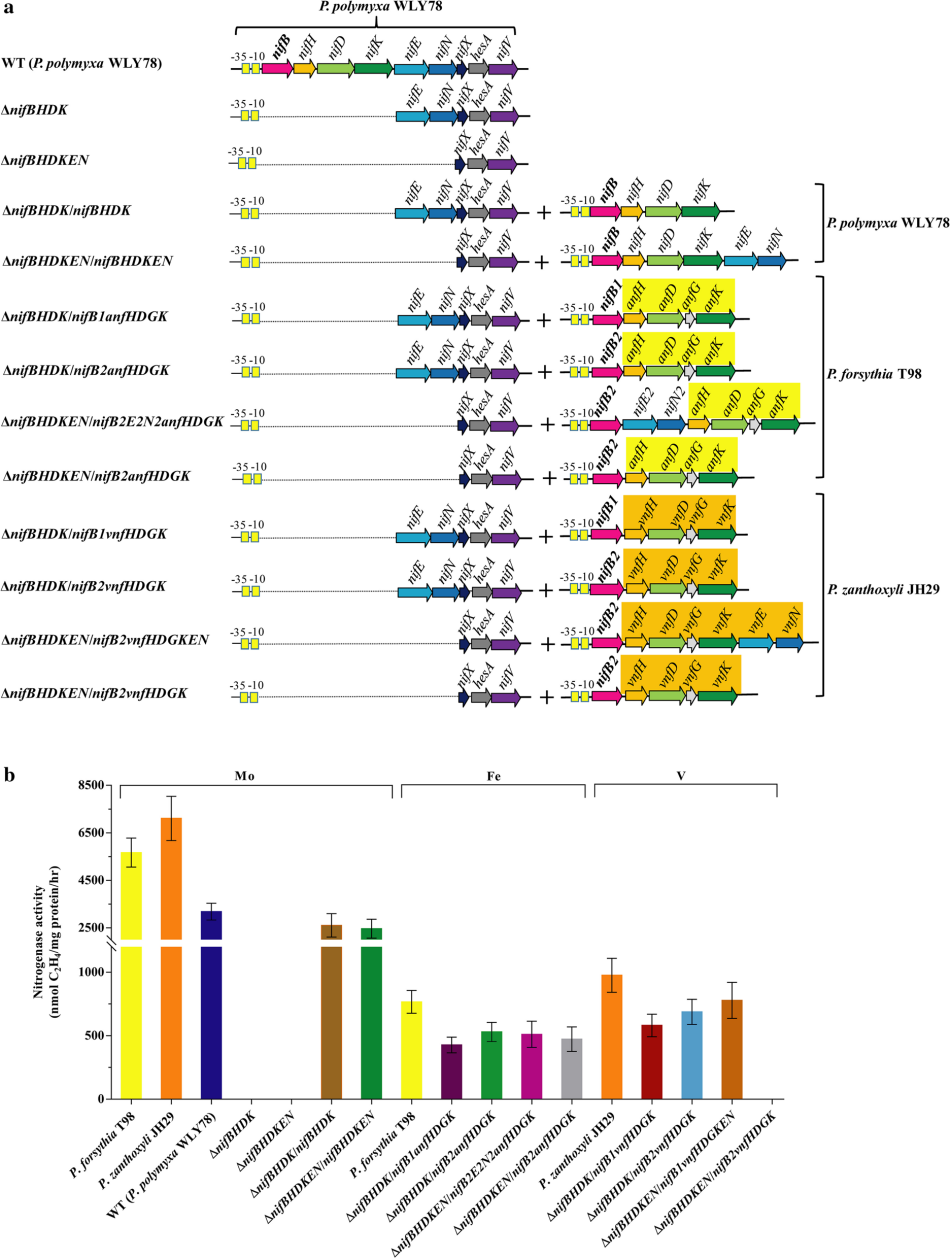
Schematic map and nitrogenase activity of the Δ*nifBHDK* and Δ*nifBHDKEN* mutants of *P. polymyxa* and the complementary strains carrying *nifB1anfHDGK*, *nifB2anfBHDGK*, *nifB2E2N2anfBHDGK* of *P. forsythia* T98, respectively and the complementary strains carrying *nifB1vnfHDGK*, *nifB2vnfHDGK*, *nifB2vnfHDGKEN* of *P. zanthoxyli* JH29, respectively. **a** Schematic map of the *P. polymyxa* Δ*nifBHDK* and *P. polymyxa* Δ*nifBHDKEN* mutants and the complementary strains. **b** The nitrogenase activity of the *P. polymyxa* Δ*nifBHDK* and *P. polymyxa* Δ*nifBHDKEN* mutants and the complementary strains. Activity was measured by acetylene reduction assay. The complementary strains carrying *nifB1anfHDGK*, *nifB2anfBHDGK* and *nifB2E2N2anfBHDGK* were cultivated in Fe-dependent conditions. The complementary strains carrying *nifB1vnfHDGK*, *nifB2vnfHDGK* and *nifB2vnfHDGKEN* were cultivated in V-dependent conditions. Error bars indicate the SD observed from at least three independent experiments

In order to investigate whether the *nifB1* and *nifB2* from *P. forsythia* T98 and *P. zanthoxyli* JH29 were active in synthesis of Fe-nitrogenase and V-nitrogenases, the Δ*nifBHDK* and *ΔnifBHDKEN* mutants of *P. polymyxa* WLY78 which lost the ability to synthesize Mo-nitrogenase were constructed. As shown in Fig. 5, the *nifBHDK* and *nifBHDKEN* of *P. polymyxa* WLY78 carried in plasmid could restore the nitrogenase activity to 90% wild-type level in the complementary strain (Δ*nifBHDK/nifBHDK*) and (Δ*nifBHDKEN/nifBHDKEN*), suggesting that the mutants can be used as a host for complementation study of alternative nitrogenases.

Two new operons *nifB1anfHDGK* and *nifB2anfHDGK* of *P. forsythia* T98 under the control of the *P. polymyxa* WLY78 *nifB* promoter were constructed (Fig. 5). Each of the reconstituted *nifB1anfHDGK* and *nifB2anfHDGK* operons of *P. forsythia* T98 carried in the recombinant plasmids can enable *P. polymyxa* Δ*nifBHDK* mutant to have nitrogenase activity in medium containing Fe and lacking Mo. The data suggest that either *nifB1* or *nifB2* together with *anfHDGK* of *P. forsythia* can support synthesis of Fe-nitrogenase in the heterologous host *P. polymyxa* which originally has only Mo-nitrogenase system. Furthermore, in order to investigate whether *nifE* and *nifN* genes (designed *nifE2* and *nifN2* genes) preceding *anfHDGK* of *P. forsythia* T98 were functional, another new operon *nifB2E2N2anfHDGK* of *P. forsythia* T98 was constructed (Fig. 5). Then, *nifB2E2N2anfHDGK* and *nifB2anfHDGK* carried in the recombinant plasmids are individually used to complement Δ*nifBHDKEN* mutant of *P. polymyxa* WLY78. As shown in Fig. 5, either *nifB2E2N2anfHDGK* or *nifB2anfHDGK* can support Δ*nifBHDKEN* mutant of *P. polymyxa* WLY78 to have nitrogenase activity in medium containing Fe and lacking Mo. Like the *P. forsythia* T98 that was capable of diazotrophic growth, the reconstituted *nifB*/*anf*-complemented strains can grow in liquid media with dinitrogen as the sole nitrogen source (Fig. S2). The results indicated that that *nifEN* is not necessary for the biosynthesis and the reconstituted *anf* system composed of 8 genes (*nifBanfHDGK* of *P. forsythia* T98 and *nifXhesAnifV* of *P. polymyxa* WLY78) can support synthesis of Fe-nitrogenase to fix nitrogen.

Similarly, two new operons *nifB1vnfHDGK* and *nifB2vnfHDGK* of *P. zanthoxyli* JH29 under the control of the *nifB* promoter of *P. polymyxa* WLY78 were constructed (Fig. 5a). Each of the *nifB1vnfHDGK* and *nifB2vnfHDGK* operons of *P. zanthoxyli* JH29 carried in the recombinant plasmids can enable *P. polymyxa* Δ*nifBHDK* mutant to have nitrogenase activity in medium containing V and lacking Mo (Fig. 5b). The data suggest that either of *nifB1* or *nifB2* together with *vnfHDGK* of *P. zanthoxyli* JH29 can support synthesis of V-nitrogenase. Furthermore, a new operon comprising *nifB2* and *vnfHDGKEN* under the control of the *nifB* promoter of *P. polymyxa* WLY78 was constructed. The reconstituted operons *nifB2vnfHDGKEN* and *nifB2vnfHDGK* of *P. zanthoxyli* JH29 are individually used to complement Δ*nifBHDKEN* mutant of *P. polymyxa* WLY78. The operon *nifB2vnfHDGKEN* can effectively enable Δ*nifBHDKEN* mutant of *P. polymyxa* WLY78 to synthesize V-nitrogenase (Fig. 5). Our data demonstrate that the reconstituted *vnf* system with *vnfEN* exhibited higher nitrogenase activity compared to the reconstituted *vnf* system with *nifEN*. However, the *nifB2vnfHDGK* operon of *P. zanthoxyli* JH29 can not complement the Δ*nifBHDKEN* mutant of *P. polymyxa* WLY78, suggesting that the *vnfEN* or *nifEN* was required for the biosynthesis of VFe-co. The diazotrophic growth tests showed that all the reconstituted *nifB*/*vnf*-complemented strains excluding Δ*nifBHDKEN*/*nifB2vnfHDGK* strain grew as well as the *P. zanthoxyli* JH29 (Additional file 1: Figure S2). The results indicated that the reconstituted *vnf* system composed of 10 genes (*nifBvnfHDGK* of *P. zanthoxyli* JH29 and *nifENXhesAnifV* of *P. polymyxa* WLY78 or *nifBvnfHDGKEN* of *P. zanthoxyli* JH29 and *nifXhesAnifV* of *P. polymyxa* WLY78) can support synthesis of V-nitrogenase to fix nitrogen.

## Discussion

Most of the diazotrophs carried a single copy of *nifB*. However, our results demonstrated that 2–4 *nifB* genes were distributed in *Paenibacillus* strains having additional *nif* genes or *anf* genes or *vnf* genes. The occurrence of multiple *nifB* copies appears to be specific to diazotrophic *Paenibacillus*. In addition, the presence of *nifB1* immediately upstream of the structural genes *nifHDK* and presence of *nifB2* close to the structural genes *anfHDGK* or *vnfHDGK* also seem to characterize the genus. Our analyses have revealed that all *nifB* genes in *Paenibacillus* fall into 4 classes and their encoded products have a N-terminal SAM-radical domain linked to a C-terminal NifX-like domain. However, the NifB3 protein of *P. zanthoxyli* JH29 or *P. durus* DSM 1735 is a stand-alone SAM-radical protein which is adjacent to a NifX-like protein. To confirm the accuracy of the *nifB3* at DNA sequence level, a DNA fragment including both of the coding regions of a SAM-radical protein and a NifX-like protein was PCR amplified from *P. zanthoxyli* JH29 (Additional file 1: Figure S3). Sequence analysis have shown that the NifB3 protein of *P. zanthoxyli* JH29 is really a stand-alone SAM-radical protein that linked to a NifX-like protein. We deduce that the *nifB3* gene of *P. zanthoxyli* JH29 or *P. durus* DSM 1735 is divided to two genes: one encoding a SAM-radical protein and the other encoding a NifX-like protein during evolution. The NifB proteins with only a SAM-radical domain are distributed in some bacteria and in most archaea [21]. However, a stand-alone SAM-radical domain in the NifB3 proteins of *P. zanthoxyli* JH29 and *P. durus* DSM 1735 lacks the C-terminal Cx2CRxDAxG motif that binds an Fe-S cluster necessary for NifB-co synthesis [29]. The NifB proteins with three domain architectures comprising a NifN-like domain, SAM-radical domain and a NifX domain are widely distributed in *Clostridium* genus [21]. However, the NifB proteins with three domain architectures are not found in *Paenibacillus*, although both *Paenibacillus* and *Clostridium* are genera of the Firmicutes phylum.

The canonical NifB protein contains a SAM-radical domain and a NifX-like domain. We have found that some N_2_-fixing *Paenibacillus* strains possess NifX-like protein that shows higher sequence similarity value with the C-terminal domain of NifB compared with that of NifX protein family. These proteins with only a NifX-like domain are also found in other diazotrophs, but they were eliminated from their studies [21]. Here, the transcription and function of the *nifX*-like genes from *P. sabinae* T27, *P. forsythia* T98 and *P. zanthoxyli* JH29 are investigated. Generally, the *nifX*-like gene in *Paenibacillus* strains is linked together with *nifH* or other gene. In *P. sabinae* T27, the *nifX*-like gene is located within the *nifH nifX*-like *nifN nifE* cluster and is significantly transcribed under N_2_-fixing condition compared to non-N_2_-fixing condition (Additional file 1: Figure S4a). One possible reason is that the *nifX*-like and *nifH* were cotranscribed from a common promoter, consistent with previous studies that transcript of the *nifH* and *nifX*-like (previously called as *nifB*) increased under nitrogen fixation condition [26, 30]. However, the transcription of *nifX*-like gene linked together with *gldA* gene in *P. forsythia* T98 or *P. zanthoxyli* JH29 was not upregulated under N_2_-fixing condition than non-N_2_-fixing condition (Additional file 1: Figure S4b, c). Complementation experiments demonstrate that NifX-like proteins of *P. sabinae* T27, *P. forsythia* T98 and *P. zanthoxyli* JH29 could not resume the nitrogenase activity of *P. polymyxa* ∆*nifB* mutant (Additional file 1: Figure S4d), indicating that these NifX-like proteins can not substitute NifB. It was reported that NifX-like domain of NifB is not required for nitrogen fixation but may perform complementary functions that are beneficial for FeMo-co biosynthesis [21].

Complementation studies revealed that either NifB1 or NifB2 protein can support any type of nitrogenase activity. However, expression analysis showed that *nifB1* exhibited the greatest increase in expression under Mo-dependent N_2_-fixing condition compared to alternative N_2_-fixing condition and *nifB2* is even more induced under alternative N_2_-fixing condition compared to Mo-dependent N_2_-fixing condition. This implies that the NifB1 or NifB2 are specifically expressed under different metal conditions to support synthesis of Mo- and alternative nitrogenases in original host cell, respectively. Some reports found that 2 *nifB* genes in diazotroph genomes [18, 20], but no further work has demonstrated their transcription levels under different metal conditions. It is reported that *P. sabinae* T27, *P. zanthoxyli* JH29 and *P. forsythia* T98 exhibited high nitrogenase activities compared to *P. polymyxa* WLY78 [31]. Previous studies showed that 3 *nifH* genes of *P. sabinae* T27 are functional by complementing *K. oxytoca* Δ*nifH* mutant [32]. Our present work demonstrated that *nifB2* restored the nitrogenase activity of *P. polymyxa* WLY78 Δ*nifB* mutant. Thus, the higher nitrogenase activity exhibited by these species may be due to their additional *nif* genes.

The *nifB3* and *nifB4* were not exhibited higher transcriptional activity under N_2_-fixing condition than under non-N_2_-fixing condition, nor functionally complementing the most common and active *nifB1* copy, and in some cases, displaying sequence divergence in regions of the protein already described as critical for NifB activity. Deletion analysis in the original *Paenibacillus* strain further revealed that *nifB3* and *nifB4* were not essential to nitrogen fixation. Thus, the *nifB3* and *nifB4* genes may be not functional or their genes products were inactive in synthesis of nitrogenase. They could be pseudogenes. Since the *nifB3* and *nifB4*-encoded proteins exhibit sequence conservation with that of NifB1 and NifB2, transcription inactivity of *nifB3* and *nifB4* seems to be caused by mutations in their regulatory sequence, leading to prevent their expression.

Moreover, we extended the studies to reconstruct gene requirements for the alternative nitrogenase. Our current study has demonstrated that the reconstituted *anf* system composed of 8 genes (*nifBanfHDGK* and *nifXhesAnifV*) can support synthesis of Fe-nitrogenase to fix nitrogen in *P. polymyxa*. This is consistent with previous report that the *nifEN* is not required for the reconstruction Fe-nitrogenase in *Escherichia coli* [33]. In contrast, synthesis of V-nitrogenase is dependent on either *nifEN* or *vnfEN*. In *A. vinelandii*, NifEN can substitute for VnfEN in *vnfEN *mutants for the biosynthesis of VFe-co, but the VnfEN not NifEN is the preferred scaffold for FeV-co maturation [34, 35]. Our result also confirms that VnfEN is more effective in FeV-co biosynthesis than NifEN.

Many efforts have been directed at engineering diazotrophic eukaryotes, one of the main hurdles is achieving NifB activity. Recent studies have found that the expressed NifB from the methanogen *Methanocaldococcus infernus* in the yeast cell was in a soluble form, while the expressed NifB from *A. vinelandii* in the yeast cells formed aggregates [36, 37]. In addition, the minimal number of genes required for nitrogen fixation is also the crucial step toward this goal. The *Paenibacillus* strains has some interesting features for engineering of eukaryotic N_2_ fixation, such as minimal *nif* gene cluster and additional *nif* and *anf* or *vnf* genes. Our study may provide guidance for screening *nif* genes to sort the best candidates to generate efficient nitrogenase. Given widespread findings of terrestrial Mo limitation [38], the minimal Fe- nitrogenase and V- nitrogenase systems described here have practical potentials in engineering nitrogen fixing plants.

## Materials and methods

### Phylogenetic analysis

The 138 putative *nifB* gene sequences of the 116 N_2_-fixing *Paenibacillus* strains and 11 putative *nifB* gene sequences of 10 other diazotrophs (*Frankia* sp. EAN1pec, *Nostoc* sp. PCC7120, *Bradyrhizobium japonicum* USDA 6, *Kyrpidia spormannii* CVV65, *Clostridium kluyveri* DSM 555, *Dehalobacter* sp. CF, *A. vinelandii* DJ, *K. oxytoca* KONIH1, *Methanococcus maripaludis* S2 and *Methanosarcina acetivorans* C2A) from the NCBI RefSeq database (last accessed July 2019) are shown in Table S1. Multiple alignment of amino acid sequences was performed by ClustalW (version 2.1) [39]. A maximum-likelihood phylogenetic tree of *Paenibacillus* species was constructed using PhyML (version 3.0) software [40].

### Plasmids, strains and growth conditions

Strains and plasmids used in this work are listed in (Additional file 1: Table S2). *Paenibacillus* strains were routinely grown in LD medium (2.5 g/L NaCl, 5 g/L yeast and 10 g/L tryptone) at 30℃ with shaking under aerobic condition. For nitrogen fixation, *Paenibacillus* strains were grown in nitrogen-limited medium (0.3 g/L glutamate) under anaerobic condition. Nitrogen-limited medium used in this study contains 10.4 g/L of Na_2_HPO4, 3.4 g/L of KH_2_PO_4_, 26 mg/L of CaCl_2_·2H_2_O, 30 mg/L of MgSO_4_, 0.3 mg/L of MnSO_4_, 36 mg/L of Ferric citrate, 7.6 mg/L Na_2_MoO_4_·2H_2_O, 10 µg/L of p-aminobenzoic acid, 5 µg/L of biotin, and 4 g/L glucose, with 0.3 g/L glutamate as the nitrogen source. *Escherichia coli* JM109 was used as routine cloning host. Thermo-sensitive vector pRN5101 [27, 28] was used for gene disruption and complementation experiment in *P. polymyxa* WLY78 and *P. sabinae* T27. When appropriate, antibiotics were added in the following concentrations: 100 μg/mL ampicillin and 5 μg/mL erythromycin for maintenance of plasmids.

For diazotrophic growth, *Paenibacillus* strains and complementary strains were initially grown overnight in LD medium at 30℃. Cells were collected, washed, and resuspended in nitrogen-free medium (nitrogen-limited medium without glutamate) under N_2_ atmosphere, with initial OD_600_ of 0.3. After 48 h, OD_600_ was detected.

### Acetylene reduction assays for nitrogenase activity

Nitrogenase activity was measured by acetylene reduction assays as described previously [25]. For Mo-nitrogenase activity, *P. polymyxa* WLY78 and their derivatives were individually grown overnight in 50 mL of liquid LD media for 16 h at 30℃ with shaking at 200 rpm. The culture was collected by centrifugation, and the pellet was washed three times with sterilized water and then resuspended in a 26 mL sealed tube containing 4 mL of nitrogen-limited medium to a final OD_600_ of 0.3 to 0.5. The headspace in the tube was then evacuated and replaced with argon gas. After C_2_H_2_ (10% of the headspace volume) was injected into the test tubes, the cultures were incubated at 30 °C for 2–4 h and with shaking at 200 rpm. Then, 100 μL of gas was withdrawn through the rubber stopper with a gas tight syringe and manually injected into the gas chromatograph HP6890 to quantify ethylene production. The nitrogenase activity was expressed in nmol C_2_H_4_/mg protein/hr. To assess Fe-nitrogenase activity, Mo-starved *Paenibacillus* cells were grown in nitrogen-limited medium that was depleted of molybdenum by Schneider et al. [41]. For V-nitrogenase activity, 30 μM Na_3_VO_4_ was added to the nitrogen-limited medium to take place of Na_2_MoO_4_. All treatments were in three replicates and all the experiments were repeated three or more than three times.

### Transcription analysis

Transcription analyses of *nifB* genes were investigated by real-time quantitative PCR (RT-qPCR). *Paenibacillus sabinae* T27 was grown in nitrogen-limited medium containing Mo (Na_2_MoO4), while *P. zanthoxyli* JH29 and *P. forsythia* T98 were grown in Mo-free nitrogen-limited media containing Fe and V, respectively. For negative controls, these bacteria were individually grown in LD medium which has excess nitrogen medium to inhibit nitrogen fixation. These *Paenibacillus* strains were grown at 30℃ with shaking under anaerobic condition. The bacterial cells were harvested after cultivation for 4 h cultivation. Total RNA was extracted with Trizol (Takara Bio, Tokyo, Japan) according to the manufacturer's instructions. The integrity and size distribution of the RNA was verified by agarose gel electrophoresis, and the concentration was determined spectrophotometrically. Remove of genome DNA and synthesis of cDNA were performed using RT Prime Mix according to the manufacturer’s specifications (Takara Bio, Tokyo, Japan). Primers for *nif* genes and 16S rDNA used for RT-qPCR are listed in Additional file 1: Table S3. RT-qPCR was performed on Applied Biosystems 7500 Real-Time System and detected by the SYBR Green detection system with the following program: 95℃ for 15 min, 1 cycle; 95℃ for 10 s and 65℃ for 30 s, 40 cycles. The relative expression level was calculated using the 2^−∆∆CT^ method [42]. Each experiment was performed in triplicate.

### Construction of ∆*nifB*, ∆*nifBHDK* and ∆*nifBHDKEN* mutants of *P. polymyxa*

The *nifB*, *nifBHDK* and *nifBHDKEN* deletion mutants of *P. polymyxa* WLY78 were constructed by a homologous recombination method. The upstream (ca. 1 kb) and downstream (ca. 1.0 kb) fragments flanking the coding region of *nifB* or *nifBHDK* or *nifBHDKEN* were amplified by PCR from the genomic DNA of *P. polymyxa* WLY78 using Super-Fidelity DNA Polymerase (Vazyme Biotech Co., Ltd., Nanjing, China), respectively. The two fragments flanking coding region of *nifB* or *nifBHDK* or *nifBHDKEN* were then fused with *BamH* I digested pRN5101 vector using Gibson assembly master mix (New England Biolabs, Ipswich, USA), generating the recombinant plasmids pRDnifB, pRDnifBHDK and pRDnifBHDKEN, respectively. Then, each of these recombinant plasmids was transformed into *P. polymyxa* WLY78 as described by Wang et al*.*, [43]. Subsequently, marker-free deletion mutants (the double-crossover transformants) Δ*nifB*, Δ*nifBHDK* and Δ*nifBHDKEN* were selected from the initial Em^r^ transformants after several rounds of nonselective growth at 39 °C and then confirmed by PCR amplification and sequencing analysis. The primers used for the PCR amplifications were listed in Additional file 1: Table S3.

### Construction of plasmids for complementation of the *P. polymyxa* Δ*nifB* mutant

Here, 9 *nifB* genes from *P. sabinae* T27, *P. forsythia* T98 and *P. zanthoxyli* JH29 were used to complement the *P. polymyxa* Δ*nifB* mutant. These *nifB* genes include *nifB1*, *nifB3* and *nifB4* of *P. sabinae* T27, *nifB1*, *nifB2* and *nifB3* of *P. forsythia* T98 and *nifB1*, *nifB2* and *nifB3* of *P. zanthoxyli* JH29. The coding region of each *nifB* gene from *P. sabinae* T27, *P. forsythia* T98 and *P. zanthoxyli* JH29 and a 310 bp promoter region of *nifB* in the *nifBHDKENXhesAnifV* operon of *P. polymyxa* WLY78 were PCR amplified. Then, The PCR products of the *nifB* coding region and the promoter region were fused together with vector pRN5101 using Gibson assembly master mix, yielding the recombinant plasmid. The recombinant plasmid was transformed to *P. polymyxa* WLY78 *nifB* mutant for complementation. The primers used in fusion were listed in Additional file 1: Table S3.

### Construction of ∆*nifB1*, ∆*nifB3* and ∆*nifB4* mutants of *P. sabinae* T27 and complementation strain

Three *nifB* deletion mutants in *P. sabinae* T27 including ∆*nifB1*, ∆*nifB3* and ∆*nifB4* were constructed via homologous recombination using the suicide plasmid pRN5101 as described above. The upstream and downstream fragments flanking the coding region of *nifB1*, *nifB3* and *nifB4* were PCR amplified from the genomic DNA of *P. sabinae* T27, respectively. The primers used for deletion mutagenesis are listed in Additional file 1: Table S3. The upstream and downstream fragments of three *nifB* genes were then fused with *BamH* I -digested vector pRN5101 in Gibson assembly master mix, generating the three recombinant plasmids pRDnifB1, pRDnifB3 and pRDnifB4. Then, each of these recombinant plasmids was electroporated into *P. sabinae* T27, and the deletion mutants were screened and confirmed by PCR and sequencing.

For complementation of Δ*nifB1*, a DNA fragment carrying the *nifB1* ORF (1377 bp) and its own promoter (549 bp) was PCR amplified and then ligated to pRN5101 and then transformed to *P. sabinae* T27 Δ*nifB1*, generating the *nifB1* complemented strain *nifB1/nifB1*. The primers used here are listed in Additional file 1: Table S3.

### Construction of the recombinant plasmids for complementation of the *P. polymyxa* Δ*nifBHDK* or Δ*nifBHDKEN* mutant

For construction recombinant plasmids of alternative nitrogenases in *P. polymyxa*, the coding regions of the *nifB1, nifB2*, the *anfHDGK* and *nifE2N2anfHDGK* operon were amplified from the genome of *P. forsythia* T98, respectively. Also, a 310 bp promoter region of *nifB* in the *nifBHDKENXhesAnifV* operon of *P. polymyxa* WLY78 was PCR amplified. Then, the PCR amplified promoter, *nifB1* or *nifB2* and the *anfHDGK* or *nifE2N2anfHDGK* operon were in order linked to vector pRN5101 using Gibson assembly master mix, yielding the recombinant plasmid carrying the reconstituted *nifB1anfHDGK* operon or *nifB2anfHDGK* operon or *nifB2E2N2anfHDGK* operon. The expression of *nifB1vnfHDGK* or *nifB2vnfHDGK* or *nifE2N2anfHDGK* was under control of the *P. polymyxa nifB* promoter. Finally, these plasmids were individually transformed into Δ*nifBHDK* or Δ*nifBHDKEN* mutant of *P*. *polymyxa* WLY78.

Similarly, the *nifB1*, *nifB2*, *vnfHDGK* and *vnfHDGKEN* operon were amplified from the genome of *P. zanthoxyli* JH29, respectively. A 310 bp promoter region of *nifB* in the *nifBHDKENXhesAnifV* operon of *P. polymyxa* WLY78 was PCR amplified. Then, the three fragments including the promoter, *nifB1* or *nifB2* and *vnfHDGK* or *vnfHDGKEN* operon were in order fused together with vector pRN5101 using Gibson assembly master mix, yielding the recombinant plasmid carrying the reconstituted operon *nifB1vnfHDGK* or *nifB2vnfHDGK* or *nifB2vnfHDGKEN.* The expression of *nifB1vnfHDGK* or *nifB2vnfHDGK* or *nifB2vnfHDGKEN* was under control of the *P. polymyxa nifB* promoter. Finally, these plasmids were individually transformed into Δ*nifBHDK* mutant or Δ*nifBHDKEN* of *P*. *polymyxa* WLY78.

## Supplementary Information

**Additional file 1**: **Table S1.** The *nifB *gene in diazotrophic *Paenibacillus *strains and other representative diazotrophs. **Figure S1.** Sequence alignment of 10 NifB proteins and 3 NifX-like proteins from 4 representatives of N_2_-fixing *Paenibacillus *strains (*P. polymyxa *WLY78, *P. sabinae *T27, *P. forsythia *T98 and *P. zanthoxyli *JH29). **Figure S2.** Diazotrophic growth of the Δ*nifBHDK *and Δ*nifBHDKEN *mutants of *P. polymyxa *and the complementary strains carrying *nifB1anfHDGK*, *nifB2anfBHDGK, nifB2E2N2anfBHDGK *from *P. forsythia *T98, and *nifB1vnfHDGK*, *nifB2vnfHDGK, nifB2vnfHDGKEN *from *P. zanthoxyli *JH29, respectively. **Figure S3.** Nucleotide sequence of DNA fragment containing *nifB3 *and an additional *nifX*-like in *P. zanthoxyli *JH29 and *P. durus *DSM 1735. **Figure S4.** Transcription analysis of the *nifX*-like genes and nitrogenase activities of the *P. polymyxa *Δ*nifB* complementary strains carrying *nifX*-like genes under nitrogen fixation conditions. **Table S2.** Bacterial strains and plasmids used in this study. **Table S3.** Primers used for RT-qPCR, construction of *nifB*,* nifBHDK*, *nifNBHDKEN*, *nifB1*,* nifB3*, *nifB4 *mutants and complementation strains.

## Data Availability

All data generated or analysed during this study are included in this published article and are available from the corresponding author on reasonable request.
