# Supplementary material for: Functional analysis of multiple nifB genes of Paenibacillus strains in synthesis of Mo-, Fe- and V-nitrogenases
Source: Microb Cell Fact. 2021 Jul 19;20:139. doi: 10.1186/s12934-021-01629-9 (PMC8287671; doi:10.1186/s12934-021-01629-9)
Supplement: Supplementary file 1 — Additional file 1: Table S1. The nifB gene in diazotrophic Paenibacillus strains and other representative diazotrophs. Figure S1. Sequence alignment of 10 NifB proteins and 3 NifX-like proteins from 4 representatives of N2-fixing Paenibacillus strains (P. polymyxa WLY78, P. sabinae T27, P. forsythia T98 and P. zanthoxyli JH29). Figure S2. Diazotrophic growth of the ΔnifBHDK and ΔnifBHDKEN mutants of P. polymyxa and the complementary strains carrying nifB1anfHDGK, nifB2anfBHDGK, nifB2E2N2anfBHDGK from P. forsythia T98, and nifB1vnfHDGK, nifB2vnfHDGK, nifB2vnfHDGKEN from P. zanthoxyli JH29, respectively. Figure S3. Nucleotide sequence of DNA fragment containing nifB3 and an additional nifX-like in P. zanthoxyli JH29 and P. durus DSM 1735. Figure S4. Transcription analysis of the nifX-like genes and nitrogenase activities of the P. polymyxa ΔnifB complementary strains carrying nifX-like genes under nitrogen fixation conditions. Table S2. Bacterial strains and plasmids used in this study. Table S3. Primers used for RT-qPCR, construction of nifB, nifBHDK, nifNBHDKEN, nifB1, nifB3, nifB4 mutants and complementation strains. [file 12934_2021_1629_MOESM1_ESM.docx]

Functional Analysis of Multiple *nifB* Genes of *Paenibacillus* Strains in Synthesis of Mo-, Fe- and V-Nitrogenases

Qin Li, Haowei Zhang, Liqun Zhang, Sanfeng Chen^*^

*Correspondence: chensf@cau.edu.cn

^1^State Key Laboratory for Agrobiotechnology, College of Biological Sciences, China Agricultural University, Beijing 100193, P.R. China

^2^Key Laboratory of Pest Monitoring and Green Management, Ministry of Agriculture and Rural Affairs, and College of Plant Protection, China Agricultural University, Beijing 100193, P.R. China

# Additional file 1

| Table S1. The nifB gene in diazotrophic Paenibacillus strains and other representative diazotrophs. | | | | | | |
| --- | --- | --- | --- | --- | --- | --- |
| **Species** | **types of nitrogenases** | ***nifB*** gene copies | **Gene location** | **Protein** | **Length (AA)** | **NifB Domain Architecture** |
| *Paenibacillus polymyxa* WLY78 | Mo-nitrogenase | 1 | B439_RS0108420 | NifB | 499 | SAM-radical and NifX-like |
| *Paenibacillus polymyxa* 1-43 | Mo-nitrogenase | 1 | L695_RS0111590 | NifB | 505 | SAM-radical and NifX-like |
| *Paenibacillus polymyxa* TD94 | Mo-nitrogenase | 1 | L694_RS0106700 | NifB | 499 | SAM-radical and NifX-like |
| *Paenibacillus beijingensis* 1-18 | Mo-nitrogenase | 1 | L693_RS0110395 | NifB | 489 | SAM-radical and NifX-like |
| *Paenibacillus* sp. Aloe-11 | Mo-nitrogenase | 1 | WG8_RS05115 | NifB | 489 | SAM-radical and NifX-like |
| *Paenibacillus maysiensis* SX-49 | Mo-nitrogenase | 1 | L696_RS0107555 | NifB | 490 | SAM-radical and NifX-like |
| *Paenibacillus terrae* HPL-003 | Mo-nitrogenase | 1 | HPL003_RS12860 | NifB | 499 | SAM-radical and NifX-like |
| *Paenibacillus massiliensis* T7 | Mo-nitrogenase | 1 | L690_RS0122625 | NifB | 492 | SAM-radical and NifX-like |
| *Paenibacillus sonchi* X19-5 | Mo-nitrogenase | 1 | IC30_RS15055 | NifB | 456 | SAM-radical and NifX-like |
| *Paenibacillus graminis* RSA19 | Mo-nitrogenase | 1 | L688_RS0103885 | NifB | 456 | SAM-radical and NifX-like |
| *Paenibacillus zanthoxyli* JH29 | Mo-nitrogenase and V-nitrogenase | 3 | L691_RS122320 | NifB1 | 458 | SAM-radical and NifX-like |
|  |  |  | L691_RS0124885 | NifB2 | 442 | SAM-radical and NifX-like |
|  |  |  | L691_RS31560 | NifB3 | 193 | SAM-radical |
| *Paenibacillus durus* ATCC 35681 | Mo-nitrogenase and V-nitrogenase | 3 | VK70_RS16330 | NifB1 | 458 | SAM-radical and NifX-like |
|  |  |  | VK70_RS02220 | NifB1 | 458 | SAM-radical and NifX-like |
|  |  |  | VK70_RS07085 | NifB2 | 442 | SAM-radical and NifX-like |
| *Paenibacillus durus* DSM 1735 | Mo-nitrogenase and V-nitrogenase | 4 | PDUR_RS06165 | NifB1 | 458 | SAM-radical and NifX-like |
|  |  |  | PDUR_RS20310 | NifB1 | 458 | SAM-radical and NifX-like |
|  |  |  | PDUR_RS11275 | NifB2 | 442 | SAM-radical and NifX-like |
|  |  |  | PDUR_RS27360 | NifB3 | 223 | SAM-radical |
| *Paenibacillus sophorae* S27 | Mo-nitrogenase and Fe-nitrogenase | 4 | BMX71_RS16155 | NifB1 | 458 | SAM-radical and NifX-like |
|  |  |  | BMX71_RS26280 | NifB1 | 458 | SAM-radical and NifX-like |
|  |  |  | BMX71_RS24025 | NifB2 | 438 | SAM-radical and NifX-like |
|  |  |  | BMX71_RS13575 | NifB3 | 456 | SAM-radical and NifX-like |
| *Paenibacillus forsythiae* T98 | Mo-nitrogenase and Fe-nitrogenase | 3 | L692_RS0112195 | NifB1 | 458 | SAM-radical and NifX-like |
|  |  |  | L692_RS0102435 | NifB2 | 437 | SAM-radical and NifX-like |
|  |  |  | L692_RS0109925 | NifB3 | 439 | SAM-radical and NifX-like |
| *Paenibacillus stellifer* DSM 14472 | Mo-nitrogenase and Fe-nitrogenase | 3 | PSTEL_RS20185 | NifB1 | 458 | SAM-radical and NifX-like |
|  |  |  | PSTEL_RS10895 | NifB2 | 441 | SAM-radical and NifX-like |
|  |  |  | PSTEL_RS16355 | NifB3 | 451 | SAM-radical and NifX-like |
| *Paenibacillus borealis* DSM 13188 | Mo-nitrogenase and Fe-nitrogenase | 3 | PBOR_RS28185 | NifB1 | 455 | SAM-radical and NifX-like |
|  |  |  | PBOR_RS13520 | NifB2 | 427 | SAM-radical and NifX-like |
|  |  |  | PBOR_RS19865 | NifB4 | 444 | SAM-radical and NifX-like |
| *Paenibacillus riograndensis* SBR5 | Mo-nitrogenase and Fe-nitrogenase | 2 | PRIO_RS27280 | NifB1 | 456 | SAM-radical and NifX-like |
|  |  |  | PRIO_RS19200 | NifB4 | 448 | SAM-radical and NifX-like |
| *Paenibacillus sabinae* T27 | Mo-nitrogenase | 3 | PSAB_RS18790 | NifB1 | 458 | SAM-radical and NifX-like |
|  |  |  | PSAB_RS15420 | NifB3 | 439 | SAM-radical and NifX-like |
|  |  |  | PSAB_RS15565 | NifB4 | 446 | SAM-radical and NifX-like |
| *Paenibacillus* rhizophilus 7197 | Mo-nitrogenase | 3 | EH198_RS08510 | NifB1 | 458 | SAM-radical and NifX-like |
|  |  |  | EH198_RS00985 | NifB3 | 439 | SAM-radical and NifX-like |
|  |  |  | EH198_RS00790 | NifB4 | 446 | SAM-radical and NifX-like |
| *Paenibacillus* sp. IHB B 3415 | Mo-nitrogenase | 2 | QW71_RS11630 | NifB1 | 455 | SAM-radical and NifX-like |
|  |  |  | QW71_RS23585 | NifB4 | 433 | SAM-radical and NifX-like |
| *Paenibacillus riograndensis* CAS34 | Mo-nitrogenase | 1 | AMQ84_RS18350 | NifB | 456 | SAM-radical and NifX-like |
| *Paenibacillus jilunlii* CGMCC 1.10239 | Mo-nitrogenase | 1 | BLQ82_RS19255 | NifB | 456 | SAM-radical and NifX-like |
| *Paenibacillus graminis* DSM 15220 | Mo-nitrogenase | 1 | PGRAT_RS25205 | NifB | 456 | SAM-radical and NifX-like |
| *Paenibacillus borealis* FSL H7 0744 | Mo-nitrogenase and Fe-nitrogenase | 1 | BSK56_RS12470 | NifB | 455 | SAM-radical and NifX-like |
| *Paenibacillus jilunlii* DSM 23019 | Mo-nitrogenase | 1 | AML91_RS13010 | NifB | 456 | SAM-radical and NifX-like |
| *Paenibacillus odorifer* DSM 15391 | Mo-nitrogenase | 1 | PODO_RS24355 | NifB | 458 | SAM-radical and NifX-like |
| *Paenibacillus odorifer* CBA7130 | Mo-nitrogenase | 1 | CD191_RS24590 | NifB | 458 | SAM-radical and NifX-like |
| *Paenibacillus odorifer* VTT E133288 | Mo-nitrogenase | 1 | CA596_RS12090 | NifB | 458 | SAM-radical and NifX-like |
| *Paenibacillus odorifer* FSL H3-0287 | Mo-nitrogenase | 1 | BSK67_RS21825 | NifB | 458 | SAM-radical and NifX-like |
| *Paenibacillus odorifer* FSL H3-0280 | Mo-nitrogenase | 1 | BJP48_RS13355 | NifB | 458 | SAM-radical and NifX-like |
| *Paenibacillus odorifer* FSL H7-0694 | Mo-nitrogenase | 1 | BSK54_RS21975 | NifB | 458 | SAM-radical and NifX-like |
| *Paenibacillus odorifer* FSL F4-0126 | Mo-nitrogenase | 1 | BSK61_RS18140 | NifB | 458 | SAM-radical and NifX-like |
| *Paenibacillus odorifer* FSL H7-0713 | Mo-nitrogenase | 1 | BSK64_RS23755 | NifB | 458 | SAM-radical and NifX-like |
| *Paenibacillus odorifer* FSL H7-0433 | Mo-nitrogenase | 1 | BSO21_RS13355 | NifB | 458 | SAM-radical and NifX-like |
| *Paenibacillus odorifer* FSL F4-0152 | Mo-nitrogenase | 1 | BJP46_RS00125 | NifB | 458 | SAM-radical and NifX-like |
| *Paenibacillus odorifer* FSL F4-0085 | Mo-nitrogenase | 1 | BSK55_RS24110 | NifB | 458 | SAM-radical and NifX-like |
| *Paenibacillus odorifer* FSL H8-0175 | Mo-nitrogenase | 1 | BSK53_RS26690 | NifB | 458 | SAM-radical and NifX-like |
| *Paenibacillus odorifer* FSL H8-0237 | Mo-nitrogenase | 1 | BSK66_RS14650 | NifB | 458 | SAM-radical and NifX-like |
| *Paenibacillus odorifer* FSL F4-0242 | Mo-nitrogenase | 1 | BSK60_RS23790 | NifB | 458 | SAM-radical and NifX-like |
| *Paenibacillus odorifer* FSL H8-0147 | Mo-nitrogenase | 1 | BSK50_RS20090 | NifB | 458 | SAM-radical and NifX-like |
| *Paenibacillus odorifer* FSL R5-0636 | Mo-nitrogenase | 1 | BJP49_RS12525 | NifB | 458 | SAM-radical and NifX-like |
| *Paenibacillus odorifer* FSL H7-0604 | Mo-nitrogenase | 1 | BJP51_RS14920 | NifB | 458 | SAM-radical and NifX-like |
| *Paenibacillus odorifer* FSL H7-0918 | Mo-nitrogenase | 1 | BSK47_RS26410 | NifB | 458 | SAM-radical and NifX-like |
| *Paenibacillus odorifer* FSL H3-0464 | Mo-nitrogenase | 1 | BSK63_RS21805 | NifB | 458 | SAM-radical and NifX-like |
| *Paenibacillus odorifer* FSL J3-0159 | Mo-nitrogenase | 1 | BJP47_RS11640 | NifB | 458 | SAM-radical and NifX-like |
| *Paenibacillus odorifer* FSL J3-0153 | Mo-nitrogenase | 1 | BSK46_RS13760 | NifB | 458 | SAM-radical and NifX-like |
| *Paenibacillus odorifer* FSL H7-0718 | Mo-nitrogenase | 1 | BSK57_RS04190 | NifB | 458 | SAM-radical and NifX-like |
| *Paenibacillus odorifer* FSL F4-0077 | Mo-nitrogenase | 1 | BK121_RS17170 | NifB | 458 | SAM-radical and NifX-like |
| *Paenibacillus odorifer* FSL J3-0155 | Mo-nitrogenase | 1 | BSK48_RS23550 | NifB | 458 | SAM-radical and NifX-like |
| *Paenibacillus odorifer* FSL R5-0937 | Mo-nitrogenase | 1 | BSK59_RS19130 | NifB | 458 | SAM-radical and NifX-like |
| *Paenibacillus odorifer* FSL R5-0883 | Mo-nitrogenase | 1 | BJP50_RS12275 | NifB | 458 | SAM-radical and NifX-like |
| *Paenibacillus odorifer* FSL H3-0305 | Mo-nitrogenase | 1 | BK125_RS23300 | NifB | 458 | SAM-radical and NifX-like |
| *Paenibacillus odorifer* FSL H3-0465 | Mo-nitrogenase | 1 | BSK58_RS10665 | NifB | 458 | SAM-radical and NifX-like |
| *Paenibacillus odorifer* FSL H8-0069 | Mo-nitrogenase | 1 | BSK62_RS27015 | NifB | 458 | SAM-radical and NifX-like |
| *Paenibacillus* sp. FSL R7-0337 | Mo-nitrogenase | 1 | BK147_RS23810 | NifB | 455 | SAM-radical and NifX-like |
| *Paenibacillus* sp. FSL R5-0912 | Mo-nitrogenase | 1 | R50912_RS26650 | NifB | 455 | SAM-radical and NifX-like |
| *Paenibacillus* sp. FSL R7-0273 | Mo-nitrogenase | 1 | BK144_RS17590 | NifB | 457 | SAM-radical and NifX-like |
| *Paenibacillus* sp. FSL P4-0081 | Mo-nitrogenase | 1 | P40081_RS29670 | NifB | 455 | SAM-radical and NifX-like |
| *Paenibacillus* sp. FSL H8-237 | Mo-nitrogenase | 1 | C171_RS08925 | NifB | 458 | SAM-radical and NifX-like |
| *Paenibacillus* sp. FSL R7-0333 | Mo-nitrogenase | 1 | BK146_RS26835 | NifB | 455 | SAM-radical and NifX-like |
| *Paenibacillus* sp. FSL R7-269 | Mo-nitrogenase | 1 | C162_RS17080 | NifB | 455 | SAM-radical and NifX-like |
| *Paenibacillus* sp. FSL R7-277 | Mo-nitrogenase | 1 | C173_RS06775 | NifB | 455 | SAM-radical and NifX-like |
| *Paenibacillus* sp. FSL H7-0357 | Mo-nitrogenase and Fe-nitrogenase | 1 | H70357_RS27415 | NifB | 456 | SAM-radical and NifX-like |
| *Paenibacillus* sp. FSL H8-0259 | Mo-nitrogenase | 1 | BK132_RS32300 | NifB | 455 | SAM-radical and NifX-like |
| *Paenibacillus* sp. FSL R7-0331 | Mo-nitrogenase | 1 | R70331_RS24320 | NifB | 457 | SAM-radical and NifX-like |
| *Paenibacillus peoriae* FSL R7-0321 | Mo-nitrogenase | 1 | BK145_RS06085 | NifB | 493 | SAM-radical and NifX-like |
| *Paenibacillus peoriae* FSL R7-0131 | Mo-nitrogenase | 1 | BK143_RS05770 | NifB | 499 | SAM-radical and NifX-like |
| *Paenibacillus peoriae* HS311 | Mo-nitrogenase | 1 | ABE82_RS05125 | NifB | 499 | SAM-radical and NifX-like |
| *Paenibacillus peoriae* FSL A5-0030 | Mo-nitrogenase | 1 | BK119_RS13375 | NifB | 499 | SAM-radical and NifX-like |
| *Paenibacillus peoriae* FSL H8-0551 | Mo-nitrogenase | 1 | BK134_RS07960 | NifB | 499 | SAM-radical and NifX-like |
| *Paenibacillus peoriae* FSL J3-0120 | Mo-nitrogenase | 1 | BK135_RS11750 | NifB | 499 | SAM-radical and NifX-like |
| *Paenibacillus peoriae* IBSD35 | Mo-nitrogenase | 1 | C5G87_RS13780 | NifB | 499 | SAM-radical and NifX-like |
| *Paenibacillus polymyxa* PPOL1 | Mo-nitrogenase | 1 | PPOLYM_RS15375 | NifB | 499 | SAM-radical and NifX-like |
| *Paenibacillus polymyxa* Sb31 | Mo-nitrogenase | 1 | RE92_RS06770 | NifB | 505 | SAM-radical and NifX-like |
| *Paenibacillus polymyxa* DSM 365 | Mo-nitrogenase | 1 | EL23_RS00165 | NifB | 499 | SAM-radical and NifX-like |
| *Paenibacillus polymyxa* CFSAN034342 | Mo-nitrogenase | 1 | A7309_RS00965 | NifB | 499 | SAM-radical and NifX-like |
| *Paenibacillus polymyxa* CCI25 | Mo-nitrogenase | 1 | AZE31_RS17015 | NifB | 499 | SAM-radical and NifX-like |
| *Paenibacillus polymyxa* CR1 | Mo-nitrogenase | 1 | X809_RS04805 | NifB | 499 | SAM-radical and NifX-like |
| *Paenibacillus polymyxa* ND24 | Mo-nitrogenase | 1 | A9P44_RS02760 | NifB | 505 | SAM-radical and NifX-like |
| *Paenibacillus polymyxa* YC0573 | Mo-nitrogenase | 1 | PPYC2_RS05775 | NifB | 499 | SAM-radical and NifX-like |
| *Paenibacillus polymyxa* ATCC 15970 | Mo-nitrogenase | 1 | VK72_RS05470 | NifB | 499 | SAM-radical and NifX-like |
| *Paenibacillus polymyxa* LY214 | Mo-nitrogenase | 1 | CG775_RS23320 | NifB | 499 | SAM-radical and NifX-like |
| *Paenibacillus polymyxa* CFSAN034343 | Mo-nitrogenase | 1 | A7312_RS03335 | NifB | 499 | SAM-radical and NifX-like |
| *Paenibacillus polymyxa* J | Mo-nitrogenase | 1 | AOU00_RS17940 | NifB | 499 | SAM-radical and NifX-like |
| *Paenibacillus polymyxa* YC0136 | Mo-nitrogenase | 1 | PPYC1_RS05170 | NifB | 499 | SAM-radical and NifX-like |
| *Paenibacillus polymyxa* CFSAN034341 | Mo-nitrogenase | 1 | A7311_RS03205 | NifB | 499 | SAM-radical and NifX-like |
| *Paenibacillus jamilae* CN9 | Mo-nitrogenase | 1 | AV545_RS17090 | NifB | 505 | SAM-radical and NifX-like |
| *Paenibacillus jamilae* NS115 | Mo-nitrogenase | 1 | NS115_RS08985 | NifB | 505 | SAM-radical and NifX-like |
| *Paenibacillus* sp. UNCCL52 | Mo-nitrogenase | 1 | BR01_RS0126475 | NifB | 499 | SAM-radical and NifX-like |
| *Paenibacillus faecis* DSM 23593 | Mo-nitrogenase | 1 | FRY98_RS02990 | NifB | 456 | SAM-radical and NifX-like |
| *Paenibacillus typhae* CGMCC 1.11012 | Mo-nitrogenase and Fe-nitrogenase | 1 | BLR64_RS13650 | NifB | 457 | SAM-radical and NifX-like |
| *Paenibacillus* sp. HW567 | Mo-nitrogenase | 1 | B212_RS0102920 | NifB | 456 | SAM-radical and NifX-like |
| *Paenibacillus* sp. lzhN1 | Mo-nitrogenase | 1 | C0638_RS02715 | NifB | 505 | SAM-radical and NifX-like |
| *Paenibacillus* sp. NFR01 | Mo-nitrogenase | 1 | BMW84_RS17795 | NifB | 456 | SAM-radical and NifX-like |
| *Paenibacillus* sp. P3E | Mo-nitrogenase | 1 | A3842_RS04570 | NifB | 456 | SAM-radical and NifX-like |
| *Paenibacillus donghaensis* KCTC 13049\| | Mo-nitrogenase | 1 | B9T62_RS25535 | NifB | 458 | SAM-radical and NifX-like |
| *Paenibacillus camerounensis* G4 | Mo-nitrogenase | 1 | BN3772_RS25115 | NifB | 457 | SAM-radical and NifX-like |
| *Paenibacillus helianthi* P26E | Mo-nitrogenase | 1 | A3844_RS07550 | NifB | 456 | SAM-radical and NifX-like |
| *Paenibacillus macerans* NBRC 15307 | Mo-nitrogenase and Fe-nitrogenase | 1 | PMA02S_RS20405 | NifB | 478 | SAM-radical and NifX-like |
| *Paenibacillus macerans* NCTC6355 | Mo-nitrogenase | 1 | DYE26_RS09095 | NifB | 478 | SAM-radical and NifX-like |
| *Paenibacillus macerans* 8244 | Mo-nitrogenase | 1 | DJ90_RS19335 | NifB | 478 | SAM-radical and NifX-like |
| *Paenibacillus*sp. Cl130 | Mo-nitrogenase | 1 | BM358_RS06115 | NifB | 499 | SAM-radical and NifX-like |
| *Paenibacillus anaericanus* DSM 15890 | Mo-nitrogenase | 1 | EJP82_RS10040 | NifB | 457 | SAM-radical and NifX-like |
| *Paenibacillus thermophilus* JCM 17693 | Mo-nitrogenase | 1 | EJF30_RS19805 | NifB | 478 | SAM-radical and NifX-like |
| *Paenibacillus thermophilus* DSM 24746 | Mo-nitrogenase | 1 | EIP94_RS19810 | NifB | 478 | SAM-radical and NifX-like |
| *Paenibacillus kribbensis* 6hRe76 | Mo-nitrogenase | 1 | BAZ21_RS06725 | NifB | 489 | SAM-radical and NifX-like |
| *Paenibacillus kribbensis* AM49 | Mo-nitrogenase | 1 | B4V02_RS20050 | NifB | 489 | SAM-radical and NifX-like |
| *Paenibacillus* sp. P32E | Mo-nitrogenase | 1 | A3848_RS06580 | NifB | 456 | SAM-radical and NifX-like |
| *Paenibacillus* sp. BK673 | Mo-nitrogenase | 1 | EV295_RS11985 | NifB | 492 | SAM-radical and NifX-like |
| *Paenibacillus wynnii* DSM 18334 | Mo-nitrogenase | 1 | PWYN_RS27195 | NifB | 459 | SAM-radical and NifX-like |
| *Paenibacillus panacisoli* DSM 21345 | Mo-nitrogenase | 1 | K331_RS0114395 | NifB | 492 | SAM-radical and NifX-like |
| *Paenibacillus antibioticophila* GD11 | Mo-nitrogenase | 1 | TX85_RS19765 | NifB | 485 | SAM-radical and NifX-like |
| *Paenibacillus* sp. P46E | Mo-nitrogenase | 1 | A3849_RS11890 | NifB | 456 | SAM-radical and NifX-like |
| *Paenibacillus rubinfantis* MT18 | Mo-nitrogenase | 1 | BN3277_RS12830 | NifB | 481 | SAM-radical and NifX-like |
| *Paenibacillus* oralis KCOM 3021 | Mo-nitrogenase | 1 | EHV15_RS27475 | NifB | 485 | SAM-radical and NifX-like |
| *Azotobacter vinelandii* DJ | Mo-nitrogenase, V-nitrogenase and Fe-nitrogenase | 1 | AVIN_RS23335 | NifB | 503 | SAM-radical and NifX-like |
| *Bradyrhizobium* japonicum USDA 6 | Mo-nitrogenase | 1 | BJ6T_RS38580 | NifB | 519 | SAM-radical and NifX-like |
| *Clostridium* kluyveri DSM 555 | Mo-nitrogenase and V-nitrogenase | 2 | CKL_RS05100 | NifB | 423 | SAM-radical and NifX-like |
|  |  |  | CKL_RS08590 | NifB | 451 | SAM-radical and NifX-like |
| *Dehalobacter* sp. CF | Mo-nitrogenase | 1 | DCF50_RS09865 | NifB | 294 | SAM radical |
| *Kyrpidia spormannii* EA-1 | Mo-nitrogenase | 1 | CVV65_RS08090 | NifB | 483 | SAM-radical and NifX-like |
| *Klebsiella oxytoca* KONIH1 | Mo-nitrogenase | 1 | KONIH1_RS18850 | NifB | 468 | SAM-radical and NifX-like |
| *Methanosarcina acetivorans* C2A | Mo-nitrogenase, V-nitrogenase and Fe-nitrogenase | 1 | MA_RS21900 | NifB | 323 | SAM radical |
| *Frankia* sp. EAN1pec | Mo-nitrogenase | 1 | FRANEAN1_RS34310 | NifB | 655 | SAM-radical and NifX-like |
| *Nostoc* sp. PCC 7120 | Mo-nitrogenase | 1 | PCC7120DELTA_RS09380 | NifB | 475 | SAM-radical and NifX-like |
| *Methanococcus maripaludis* S2 | Mo-nitrogenase | 1 | MMP_RS03455 | NifB | 297 | SAM radical |

| Table S2. Bacterial strains and plasmids used in this study | | |
| --- | --- | --- |
| **Strain or plasmid** | **Description** | **Source or reference** |
| **Strains** | | |
| *Paenibacillus polymyxa* WLY78 | Wild-type strain | Laboratory stock |
| Δ*nifB* | *nifB* in-frame deletion mutant of *P. polymyxa* WLY78 | This study |
| Δ*nifB/nifB* | Complementation strain of *P. polymyxa* WLY78 Δ*nifB* with *P. polymyxa* WLY78 *nifB* carried in plasmid pC78*nifB* | This study |
| *Paenibacillus sabinae* T27 | Wild-type strain | Laboratory stock |
| Δ*nifB1* | *nifB1* in-frame deletion mutant of *P. sabinae* T27 | This study |
| Δ*nifB3* | *nifB3* in-frame deletion mutant of *P. sabinae* T27 | This study |
| Δ*nifB4* | *nifB4* in-frame deletion mutant of *P. sabinae* T27 | This study |
| Δ*nifB1/nifB1* | Complementation strain of *P. sabinae* T27 Δ*nifB1* with *P. sabinae* T27 *nifB1* carried in plasmid pC*nifB1* | This study |
| Δ*nifB/*T27 *nifB1* | Complementation strain of *P. polymyxa* WLY78 Δ*nifB* with *P. sabinae* T27 *nifB1* carried in plasmid pC27*nifB1* | This study |
| Δ*nifB1/*T27 *nifB3* | Complementation strain of *P. polymyxa* WLY78 Δ*nifB* with *P. sabinae* T27 *nifB3* carried in plasmid pC27*nifB3* | This study |
| Δ*nifB1/*T27 *nifB4* | Complementation strain of *P. polymyxa* WLY78 Δ*nifB* with *P. sabinae* T27 *nifB4* carried in plasmid pC27*nifB4* | This study |
| Δ*nifB1/*T27 *nifX-like* | Complementation strain of *P. polymyxa* WLY78 Δ*nifB* with *P. sabinae* T27 *nifX-like* carried in plasmid pC27*nifB5* | This study |
| *Paenibacillus forsythia* T98 | Wild-type strain | Laboratory stock |
| Δ*nifB/*T98 *nifB1* | Complementation strain of *P. polymyxa* WLY78 Δ*nifB* with *P. forsythia* T98 *nifB1* carried in plasmid pC98*nifB1* | This study |
| Δ*nifB/*T98 *nifB2* | Complementation strain of *P. polymyxa* WLY78 Δ*nifB* with *P. forsythia* T98 *nifB2* carried in plasmid pC98*nifB2* | This study |
| Δ*nifB/*T98 *nifB3* | Complementation strain of *P. polymyxa* WLY78 Δ*nifB* with *P. forsythia* T98 *nifB3* carried in plasmid pC98*nifB3* | This study |
| Δ*nifB/*T98 *nifX-like* | Complementation strain of *P. polymyxa* WLY78 Δ*nifB* with *P. forsythia* T98 *nifX-like* carried in plasmid pC98*nifB4* | This study |
| *Paenibacillus zanthoxyli* JH29 | Wild-type strain | This study |
| Δ*nifB/*JH29 *nifB1* | Complementation strain of *P. polymyxa* WLY78 Δ*nifB* with *P. zanthoxyli* JH29 *nifB1* carried in plasmid pC29*nifB1* | This study |
| Δ*nifB/*JH29 *nifB2* | Complementation strain of *P. polymyxa* WLY78 Δ*nifB* with *P. zanthoxyli* JH29 *nifB2* carried in plasmid pC29*nifB2* | This study |
| Δ*nifB/*JH29 *nifB3* | Complementation strain of *P. polymyxa* WLY78 Δ*nifB* with *P. zanthoxyli* JH29 *nifB3* carried in plasmid pC98*nifB3* | This study |
| Δ*nifB/*JH29 *nifX-*like | Complementation strain of *P. polymyxa* WLY78 Δ*nifB* with *P. zanthoxyli* JH29 *nifX*-like carried in plasmid pC98*nifB4* | This study |
| Δ*nifBHDK* | *nifBHDK* in-frame deletion mutant of *P. polymyxa* WLY78 | This study |
| Δ*nifBHDK/nifBHDK* | Complementation strain of *P. polymyxa* WLY78 Δ*nifBHDK* with *P. polymyxa* WLY78 *nifBHDK* carried in plasmid pC78*nifBHDK* | This study |
| Δ*nifBHDKEN* | *nifBHDKEN* in-frame deletion mutant of *P. polymyxa* WLY78 | This study |
| Δ*nifBHDKEN/nifBHDKEN* | Complementation strain of *P. polymyxa* WLY78 Δ*nifBHDKEN* with *P. polymyxa* WLY78 *nifBHDKEN* carried in plasmid pC78*nifBHDKEN* | This study |
| Δ*nifBHDK/nifB1anfHDGK* | Reconstruction Fe-Fe system in *P. polymyxa* WLY78 Δ*nifBHDK* carried in plasmid pR98*nifB1anfHDGK* | This study |
| Δ*nifBHDK/nifB2anfHDGK* | Reconstruction Fe-Fe system in *P. polymyxa* WLY78 Δ*nifBHDK* carried in plasmid pR98*nifB2anfHDGK* | This study |
| Δ*nifBHDKEN/nifB2E2N2anfHDGK* | Reconstruction Fe-Fe system in *P. polymyxa* WLY78 Δ*nifBHDKEN* carried in plasmid pR98*nifB2E2N2anfHDGK* | This study |
| Δ*nifBHDKEN/nifBanfHDGK* | Reconstruction Fe-Fe system in *P. polymyxa* WLY78 Δ*nifBHDKEN* carried in plasmid pR98*nifB2anfHDGK* | This study |
| Δ*nifBHDGK/nifB1vnfHDGK* | Reconstruction V-Fe system in *P. polymyxa* WLY78 Δ*nifBHDK* carried in plasmid pR29 *nifB1vnfHDGK* | This study |
| Δ*nifBHDGK/nifB2vnfHDGK* | Reconstruction V-Fe system in *P. polymyxa* WLY78 Δ*nifBHDK* carried in plasmid pR29 *nifB2vnfHDGK* | This study |
| Δ*nifBHDGKEN/nifB2vnfHDGKEN* | Reconstruction V-Fe system in *P. polymyxa* WLY78 Δ*nifBHDKEN* carried in plasmid pR29*nifB2vnfHDGKEN* | This study |
| Δ*nifB1HDGKEN/nifB2vnfHDGK* | Reconstruction V-Fe system in *P. polymyxa* WLY78 Δ*nifBHDKEN* carried in plasmid pR29 *nifB2vnfHDGK* | This study |
| **Plasmids** | | |
| pRN5101 | Temperature-sensitive *E. coli-Bacillus* shuttle vector, Amp^R^Em^R^ | (Villafane *et al*., 1987) |
| pRD*nifB* | *P. polymyxa* WLY78 Δ*nifB* deletion vector based on pRN5101 | This study |
| pC*nifB* | *P. polymyxa* WLY78 Δ*nifB* complemented vector with *P. polymyxa* WLY78 *nifB* in pRN5101 | This study |
| pRD*nifB1* | *P. sabinae* T27 Δ*nifB1* deletion vector based on pRN5101 | This study |
| pRD*nifB3* | *P. sabinae* T27 Δ*nifB3* deletion vector based on pRN5101 | This study |
| pRD*nifB4* | *P. sabinae* T27 Δ*nifB4* deletion vector based on pRN5101 | This study |
| pC*nifB1* | *P. sabinae* T27 Δ*nifB1* complemented vector with *P. sabinae* T27 *nifB1* in pRN5101 | This study |
| pC27*nifB1* | *P. polymyxa* WLY78 Δ*nifB* complemented vector with *P. sabinae* T27 *nifB1* in pRN5101 | This study |
| pC27*nifB3* | *P. polymyxa* WLY78 Δ*nifB* complemented vector with *P. sabinae* T27 *nifB3* in pRN5101 | This study |
| pC27*nifB4* | *P. polymyxa* WLY78 Δ*nifB* complemented vector with *P. sabinae* T27 *nifB4* in pRN5101 | This study |
| pC27*nifX-like* | *P. polymyxa* WLY78 Δ*nifB* complemented vector with *P. sabinae* T27 *nifX-like* in pRN5101 | This study |
| pC98*nifB1* | *P. polymyxa* WLY78 Δ*nifB* complemented vector with *P. forsythia* T98 *nifB1* in pRN5101 | This study |
| pC98*nifB2* | *P. polymyxa* WLY78 Δ*nifB* complemented vector with *P. forsythia* T98 *nifB2* in pRN5101 | This study |
| pC98*nifB3* | *P. polymyxa* WLY78 Δ*nifB* complemented vector with *P. forsythia* T98 *nifB3* in pRN5101 | This study |
| pC98*nifX-like* | *P. polymyxa* WLY78 Δ*nifB* complemented vector with *P. forsythia* T98 *nifX-like* in pRN5101 | This study |
| pC29*nifB1* | *P. polymyxa* WLY78 Δ*nifB* complemented vector with *P. zanthoxyli* JH29 *nifB1* in pRN5101 | This study |
| pC29*nifB2* | *P. polymyxa* WLY78 Δ*nifB* complemented vector with *P. zanthoxyli* JH29 *nifB2* in pRN5101 | This study |
| pC29*nifB3* | *P. polymyxa* WLY78 Δ*nifB* complemented vector with *P. zanthoxyli* JH29 *nifB3* in pRN5101 | This study |
| pC29*nifX-like* | *P. polymyxa* WLY78 Δ*nifB* complemented vector with *P. zanthoxyli* JH29 *nifX-like* in pRN5101 | This study |
| pRD78*nifBHDK* | *P. polymyxa* WLY78 Δ*nifBHDK* deletion vector based on pRN5101 | This study |
| pC78*nifBHDK* | *P. polymyxa* WLY78 Δ*nifBHDK* complemented vector with *P. polymyxa* WLY78 *nifBHDK* in pRN5101 | This study |
| pRD78*nifBHDKEN* | *P. polymyxa* WLY78 Δ*nifBHDK* deletion vector based on pRN5101 | This study |
| pC78*nifBHDKEN* | *P. polymyxa* WLY78 Δ*nifBHDKEN* complemented vector with *P. polymyxa* WLY78 *nifBHDKEN* in pRN5101 | This study |
| pR98*nifB1anfHDGK* | *P_nif_-*T98*nifB1-anfHDGK* fusion vector based on pRN5101 | This study |
| pR98*nifB2anfHDGK* | *P_nif_-*T98*nifB2-anfHDGK* fusion vector based on pRN5101 | This study |
| pR98*nifB2E2N2anfHDGK* | *P_nif_-*T98*nifB2E2N2-anfHDGK* fusion vector based on pRN5101 | This study |
| pR29*nifB1vnfHDGK* | *P_nif_-*JH29*nifB1-vnfHDGK* fusion vector based on pRN5101 | This study |
| pR29*nifB21vnfHDGK* | *P_nif_-*JH29*nifB2-vnfHDGK* fusion vector based on pRN5101 | This study |
| pR29*nifB2vnfHDGKEN* | *P_nif_-*JH29*nifB2-vnfHDGKEN* fusion vector based on pRN5101 | This study |

| Table S3. Primers used for qRT-PCR, construction of *nifB, nifBHDK* and *nifNBHDKEN* mutants, and complementation strains. | | |
| --- | --- | --- |
| **Primer name** | **Sequence** | **Location /Target** |
| 16S-F | TTTGTCGTCAGCCTCGTGTTCGTG | qRT-PCR for control (16S rDNA) |
| 16S-R | ATCCCCACCTTCCTCCGGTTTG |  |
| 27nifB1F | CGTCTTCTACCACACCGAACT | qRT-PCR for *nifB1 of P. sabinae* T27 |
| 27nifB1R | CACAACCAAGCCAACGAATA |  |
| 27nifB3F | CGAAATGCTGACCATCCCA | qRT-PCR for *nifB3 of P. sabinae* T27 |
| 27nifB3R | TTAGGCGTTGGCTGCTGC |  |
| 27nifB4F | GAGAACCCGATTATGACTAAAAAAC | qRT-PCR for *nifB4 of P. sabinae* T27 |
| 27nifB4R | CTTTGGTGTTCAGAATCAGGC |  |
| 27nifX-likeF | GACGACCACATCCAACATCAGG | qRT-PCR for *nifX-like of P. sabinae* T27 |
| 27nifX-likeR | CAGCTTCACATTGGCGCTGT |  |
| 98nifB1F | GCAATCTACGGCAAAGGGGGG | qRT-PCR for *nifB1*of *P. forsythia* T98 |
| 98nifB1R | CGAATCGCTCTTTGGGTCGC |  |
| 98nifB2F | CACAGTCATATCCATGTCCTTGG | qRT-PCR for *nifB2*of *P. forsythia* T98 |
| 98nifB2R | GTCAAATGCACTCCTGGGATTA |  |
| 98nifB3F | GTGCTTACCGCTTTCTACGG | qRT-PCR for *nifB3* of *P. forsythia* T98 |
| 98nifB3R | GCCTATCACCTCGTCGTCTC |  |
| 98nifX-likeF | CATTAAACATAAAGTGCCGCCGG | qRT-PCR for *nifX-like* of *P. forsythia* T98 |
| 98nifX-likeR | CTGGGTTTGGGAGAAGGTGCG |  |
| 29nifB1F | GAATTTAACCGAGGTTCCGGTGC | qRT-PCR for *nifB1*of *P. zanthoxyli* JH29 |
| 29nifB1R | GTTCCCTGAGCTGCAGCCCATG |  |
| 29nifB2F | TCCTGCACAGTGGAACTGAC | qRT-PCR for *nifB2*of *P. zanthoxyli* JH29 |
| 29nifB2R | TATCCGGCTTGACCTTTGAC |  |
| 29nifB3F | CTTTGACGCGACCCAGCATG | qRT-PCR for *nifB3* of *P. zanthoxyli* JH29 |
| 29nifB3R | GATCGGAGACGGATGCCATATC |  |
| 29nifX-likeF | GCATCGTAATCCGATTATCGG | qRT-PCR for *nifX-like* of *P. zanthoxyli* JH29 |
| 29nifX-likeR | AGCGAACCAAATCAACGAGCC |  |
| nifBUpF | ACGATGCGTCCGGCGTAGAGGATCCTAGGCCTGTGGACCGCAG | In-frame deletion of *P. polymyxa* WLY78 *nifB* |
| nifBUpR | TTAACTCCCTTCATTCCCTCCTCTCTACGTTATA |  |
| nifBDownF | GAGGGAATGAAGGGAGTTAAGGGCAATCATTCG |  |
| nifBDownR | GCGACCACACCCGTCCTGTGGATCCCCTCCTGCGAGCTGCGCG |  |
| 78BF | ACGATGCGTCCGGCGTAGAGGATCCGCGGAGACTATTTCCCAAAAT | Complementation of *P. polymyxa* WLY78 Δ*nifB* with *nifB* gene |
| 78BR | GCGACCACACCCGTCCTGTGGATCCATTTTCTCAGCCTCCAAAATG |  |
| pnifF | ACGATGCGTCCGGCGTAGAGGATCCGCGGAGACTATTTCCCAAAAT | Complementation of *P. polymyxa* WLY78 Δ*nifB* with  *P. sabinae* T27 *nifB1* gene |
| pnifR1 | GCTGCGGCTTCATTCATTCCCTCCTCTCTAC |  |
| 27B1F | GAGGGAATGAATGAAGCCGCAGCCGACGTC |  |
| 27B1R | GCGACCACACCCGTCCTGTGGATCCCATGATTAATCATCCCTTCGAT |  |
| pnifF | ACGATGCGTCCGGCGTAGAGGATCCGCGGAGACTATTTCCCAAAAT | Complementation of *P. polymyxa* WLY78 Δ*nifB* with  *P. sabinae* T27 *nifB3* gene |
| pnifR2 | TCAATTGTTTCCACTCATTCCCTCCTCTCTAC |  |
| 27B3F | GAGGGAATGAGTGGAAACAATTGACGCCGC |  |
| 27B3R | GCGACCACACCCGTCCTGTGGATCCTCAAATCCCTAAATCCTTG |  |
| pnifF | ACGATGCGTCCGGCGTAGAGGATCCGCGGAGACTATTTCCCAAAAT | Complementation of *P. polymyxa* WLY78 Δ*nifB* with  *P. sabinae* T27 *nifB4* gene |
| pnifR3 | TAATGATGCGCCATCATTCCCTCCTCTCTAC |  |
| 27B4F | GAGGGAATGAATGGCGCATCATTATTTTGCCCG |  |
| 27B4R | GCGACCACACCCGTCCTGTGGATCCTCACCGCGGCTGCGCAGCGGA |  |
| pnifF | ACGATGCGTCCGGCGTAGAGGATCCGCGGAGACTATTTCCCAAAAT | Complementation of *P. polymyxa* WLY78 Δ*nifB* with  *P. sabinae* T27 *nifX-like* gene |
| pnifR4 | TGCTCTGCTGCAATCATTCCCTCCTCTCTAC |  |
| 27XF | GAGGGAATGATTGCAGCAGAGCACCAGGCA |  |
| 27XR | GCGACCACACCCGTCCTGTGGATCCTCAACTAATTTGGCTATCTCTG |  |
| pnifF | ACGATGCGTCCGGCGTAGAGGATCCGCGGAGACTATTTCCCAAAAT | Complementation of *P. polymyxa* WLY78 Δ*nifB* with  *P. forsythia* T98 *nifB1*gene |
| pnifR5 | GCGGCTTCATTCATTCCCTCCTCTCTAC |  |
| 98B1F | GAGGGAATGAATGAAGCCGCAGCCGACG |  |
| 98B1R | GCGACCACACCCGTCCTGTGGATCCGGTTTCTTACTCATGAATAATC |  |
| pnifF | ACGATGCGTCCGGCGTAGAGGATCCGCGGAGACTATTTCCCAAAAT | Complementation of *P. polymyxa* WLY78 Δ*nifB* with  *P. forsythia* T98 *nifB2*gene |
| pnifR6 | TTAATTCCACTCATTCCCTCCTCTCTAC |  |
| 98B2F | GAGGGAATGAGTGGAATTAATCGGTGAC |  |
| 98B2R | GCGACCACACCCGTCCTGTGGATCCTCATTTTGTTAAACTTAAACCG |  |
| pnifF | ACGATGCGTCCGGCGTAGAGGATCCGCGGAGACTATTTCCCAAAAT | Complementation of *P. polymyxa* WLY78 Δ*nifB* with  *P. forsythia* T98 *nifB3*gene |
| pnifR7 | ATTCCGACACTCATTCCCTCCTCTCTAC |  |
| 98B3F | GAGGGAATGAGTGTCGGAATCGGCTTTG |  |
| 98B3R | GCGACCACACCCGTCCTGTGGATCCTACTGGGCAAAGTATGTTAG |  |
| pnifF | ACGATGCGTCCGGCGTAGAGGATCCGCGGAGACTATTTCCCAAAAT | Complementation of *P. polymyxa* WLY78 Δ*nifB* with  *P. forsythia* T98 *nifX-like* gene |
| pnifR8 | GATGCGCCATTCATTCCCTCCTCTCTAC |  |
| 98XF | GAGGGAATGAATGGCGCATCATTATTTTGCCCGC |  |
| 98XR | GCGACCACACCCGTCCTGTGGATCCTCACCGCTGCTGCGCAGC |  |
| pnifF | ACGATGCGTCCGGCGTAGAGGATCCGCGGAGACTATTTCCCAAAAT | Complementation of *P. polymyxa* WLY78 Δ*nifB* with *P. zanthoxyli* JH29 *nifB1* gene |
| pnifR9 | GCGGCTTCATTCATTCCCTCCTCTCTAC |  |
| 29B1F | GAGGGAATGAATGAAGCCGCAGCCGACG |  |
| 29B1R | GCGACCACACCCGTCCTGTGGATCTCATGATTAATCATCCCTTCGA |  |
| pnifF | ACGATGCGTCCGGCGTAGAGGATCCGCGGAGACTATTTCCCAAAAT | Complementation of *P. polymyxa* WLY78 Δ*nifB* with *P. zanthoxyli* JH29 *nifB2* gene |
| pnifR10 | CTGGCTCCATTCATTCCCTCCTCTCTAC |  |
| 29B2F | GAGGGAATGAATGGAGCCAGCGTTGGCG |  |
| 29B2R | GCGACCACACCCGTCCTGTGGATCCTTAATCTCTCTTGAAATAGCC |  |
| pnifF | ACGATGCGTCCGGCGTAGAGGATCCGCGGAGACTATTTCCCAAAAT | Complementation of *P. polymyxa* WLY78 Δ*nifB* with *P. zanthoxyli* JH29 *nifB3* gene |
| pnifR11 | GATTAATTCCACTCATTCCCTCCTCTCTAC |  |
| 29B3F | GATTAATTCCACGTGGAATTAATCGGTGACGA |  |
| 29B3R | GCGACCACACCCGTCCTGTGGATCCTTCTGACGGACATCATCTTG |  |
| pnifF | ACGATGCGTCCGGCGTAGAGGATCCGCGGAGACTATTTCCCAAAAT | Complementation of *P. polymyxa* WLY78 Δ*nifB* with *P. zanthoxyli* JH29 *nifX*-*like* gene |
| pnifR12 | CGTTCGCCAATCATTCCCTCCTCTCTAC |  |
| 29XF | GAGGGAATGATTGGCGAACGAATCACGC |  |
| 29XR | GCGACCACACCCGTCCTGTGGATCCTTAACGCCGGGAATCAGC |  |
| nifB1UpF | ACGATGCGTCCGGCGTAGAGGATCCCTTGTTTCCTCAGAAAGG | In-frame deletion of *P. sabinae* T27 *nifB1* |
| nifB1UpR | CCTTCGATATTTTTTCCCACCTCCTTAATG |  |
| nifB1DownF | GTGGGAAAAAATATCGAAGGGATGATTAATC |  |
| nifB1DownR | GCGACCACACCCGTCCTGTGGATCCTCTATTGTTACGCGAATTTC |  |
| nifB3UpF | ACGATGCGTCCGGCGTAGAGGATCCCTTTTCCCCGATCCCGAAG | In-frame deletion of *P. sabinae* T27 *nifB3* |
| nifB3UpR | TCCTGGACCGCTCGTCGTAACAAGGATGG |  |
| nifB3DownF | TTACGACGAGCGGTCCAGGAAGATTAAGC |  |
| nifB3DownR | GCGACCACACCCGTCCTGTGGATCCGGACATGCTGAAGCCCAAAC |  |
| nifB4UpF | ACGATGCGTCCGGCGTAGAGGATCCTGCCTGAGAGGCCTCCAC | In-frame deletion of *P. sabinae* T27 *nifB4* |
| nifB4UpR | CAGCAGAGCAATTAGTTGACGGATCAACCGGAAG |  |
| nifB4DownF | GTCAACTAATTGCTCTGCTGCAAAACATTCAC |  |
| nifB4DownR | GCGACCACACCCGTCCTGTGGATCGGCTCGAACGGCTACAAAG |  |
| 27pB1F | ACGATGCGTCCGGCGTAGAGGATCCTCAACGACGGAAGCTCAG | Complementation of *P. sabinae* T27 Δ*nifB1* with *nifB1* gene |
| 27B1R | GCGACCACACCCGTCCTGTGGATCCCATGATTAATCATCCCTTCGAT |  |
| nifBUpF | ACGATGCGTCCGGCGTAGAGGATCCTAGGCCTGTGGACCGCAG | In-frame deletion of *P. polymyxa* WLY78 *nifBHDK* |
| nifBKUpR | CCGGAGCCTGTCCATTCATTCCCTCCTCTCTAC |  |
| nifBKDownF | AATGAATGGACAGGCTCCGGATCATAGC |  |
| nifBKDownR | GCGACCACACCCGTCCTGTGGATCCGCACTGCCCGCTTTCCTTTC |  |
| nifBUpF | ACGATGCGTCCGGCGTAGAGGATCCTAGGCCTGTGGACCGCAG | In-frame deletion of *P. polymyxa* WLY78 *nifBHDKEN* |
| nifBNUpR | TTGTTCACCATTCATTCCCTCCTCTCTACGTTATAT |  |
| nifBNDownF | AGGAGGGAATGAATGGTGAACAAAGTAGGCAATG |  |
| nifBNDownR | GCGACCACACCCGTCCTGTGGATCCGTACATGGCC GCTTCTATCA |  |
| pnifF | ACGATGCGTCCGGCGTAGAGGATCCGCGGAGACTATTTCCCAAAAT | Complementation of *P. polymyxa* WLY78 Δ*nifBHDK* with *nifBHDK*gene |
| 78BHDKR | GCGACCACACCCGTCCTGTGGATCCCCAAGCTGTCAACTTCCTTC |  |
| pnifF | ACGATGCGTCCGGCGTAGAGGATCCGCGGAGACTATTTCCCAAAAT | Complementation of *P. polymyxa* WLY78 Δ*nifBHDKEN* with *nifBHDKEN* gene |
| 78BHDKENR | ACGATGCGTCCGGCGTAGAGGATCCCAAGCACGCCGTCTTCCGTCG |  |
| pnifF | ACGATGCGTCCGGCGTAGAGGATCCGCGGAGACTATTTCCCAAAAT | Complementation of *P. polymyxa* WLY78 Δ*nifBHDK* with *P. forsythia* T98 *nifB1*and *anfHDGK* |
| nifB1aR | CCAGCCTATCTCATCCCTTCGATATATTAATA |  |
| anfHKF | GAAGGGATGAGATAGGCTGGGGTTCTTG |  |
| anfHKR | GCGACCACACCCGTCCTGTGGATCCGCTACCAAACGTTCAGCAG |  |
| pnifF | ACGATGCGTCCGGCGTAGAGGATCCGCGGAGACTATTTCCCAAAAT | Complementation of *P. polymyxa* WLY78 Δ*nifBHDK* with *P. forsythia* T98 *nifB2* and *anfHDGK* |
| nifB2aR | CCAGCCTATCCTACTGGGCAAAGTATGTTAG |  |
| anfHKF2 | TGCCCAGTAGGATAGGCTGGGGTTCTTG |  |
| anfHKR | GCGACCACACCCGTCCTGTGGATCCGCTACCAAACGTTCAGCAG |  |
| pnifF | ACGATGCGTCCGGCGTAGAGGATCCGCGGAGACTATTTCCCAAAAT | Complementation of *P. polymyxa* WLY78 Δ*nifBHDKEN* with *P. forsythia* T98 *nifB2E2N2* and *anfHDGK* |
| nifB2E2N2aR | GACGCATAAATGAATGAGAGGAGCTATTTCAA |  |
| anfHKF3 | GCTCCTCTCATTCATTTATGCGTCCCCCTTTC |  |
| anfHKR | GCGACCACACCCGTCCTGTGGATCCGCTACCAAACGTTCAGCAG |  |
| pnifF | ACGATGCGTCCGGCGTAGAGGATCCGCGGAGACTATTTCCCAAAAT | Complementation of *P. polymyxa* WLY78 Δ*nifBHDK* with *P. zanthoxyli* JH29 *nifB1* and *vnfHDGK* |
| nifB1vR | TCCTATCCGTTCATCCCTTCGATATATTA |  |
| vnfHKF | GAAGGGATGAACGGATAGGAAAGTGGTTG |  |
| vnfHKR | GCGACCACACCCGTCCTGTGGATCCTTAATCTCTCTTGAAATAGCC |  |
| pnifF | ACGATGCGTCCGGCGTAGAGGATCCGCGGAGACTATTTCCCAAAAT | Complementation of *P. polymyxa* WLY78 Δ*nifBHDK* with *P. zanthoxyli* JH29 *nifB2* and *vnfHDGK* |
| nifB2vR | TCCTATCCGTTTAATCTCTCTTGAAATAGC |  |
| vnfHKF2 | GAGAGATTAAACGGATAGGAAAGTGGTTG |  |
| vnfHKR | GCGACCACACCCGTCCTGTGGATCCTTGAAAGCTTCTTACCAGAC |  |
| pnifF | ACGATGCGTCCGGCGTAGAGGATCCGCGGAGACTATTTCCCAAAAT | Complementation of *P. polymyxa* WLY78 Δ*nifBHDK* with *P. zanthoxyli* JH29 *nifB2* and *vnfHDGKEN* |
| nifB2vR | TCCTATCCGTTTAATCTCTCTTGAAATAGC |  |
| vnfHKF2 | GAGAGATTAAACGGATAGGAAAGTGGTTG |  |
| vnfHNR | GCGACCACACCCGTCCTGTGGATCCTCACGCAAATTGCTCCCAATTG |  |

**Additional figures**


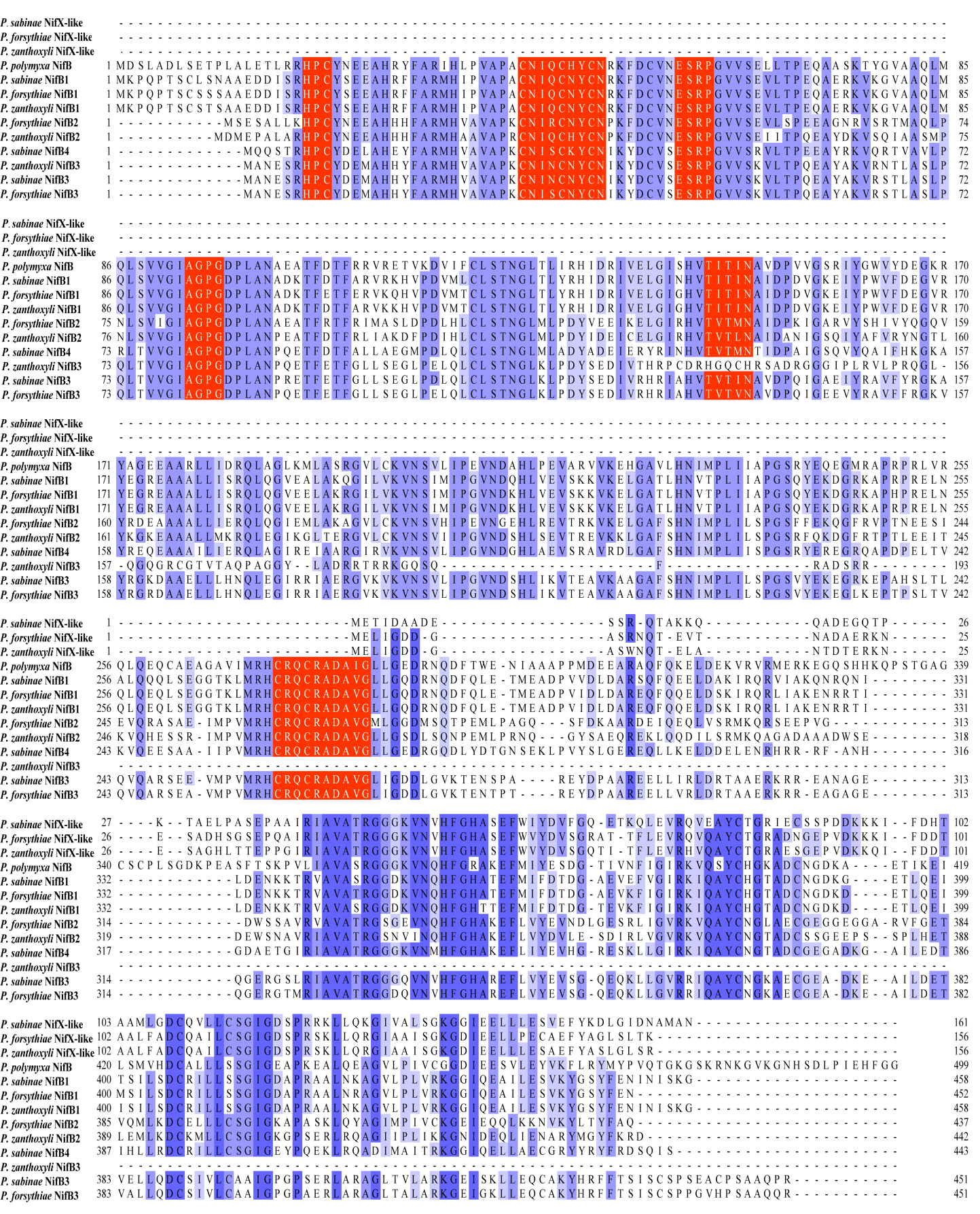


Figure S1. Sequence alignment of 10 NifB proteins and 3 NifX-like proteins from 4 representatives of N_2_-fixing *Paenibacillus* strains (*P. polymyxa* WLY78, *P. sabinae* T27, *P. forsythia* T98 and *P. zanthoxyli* JH29). Conserved motifs within the SAM-radical domain are highlighted with red background.


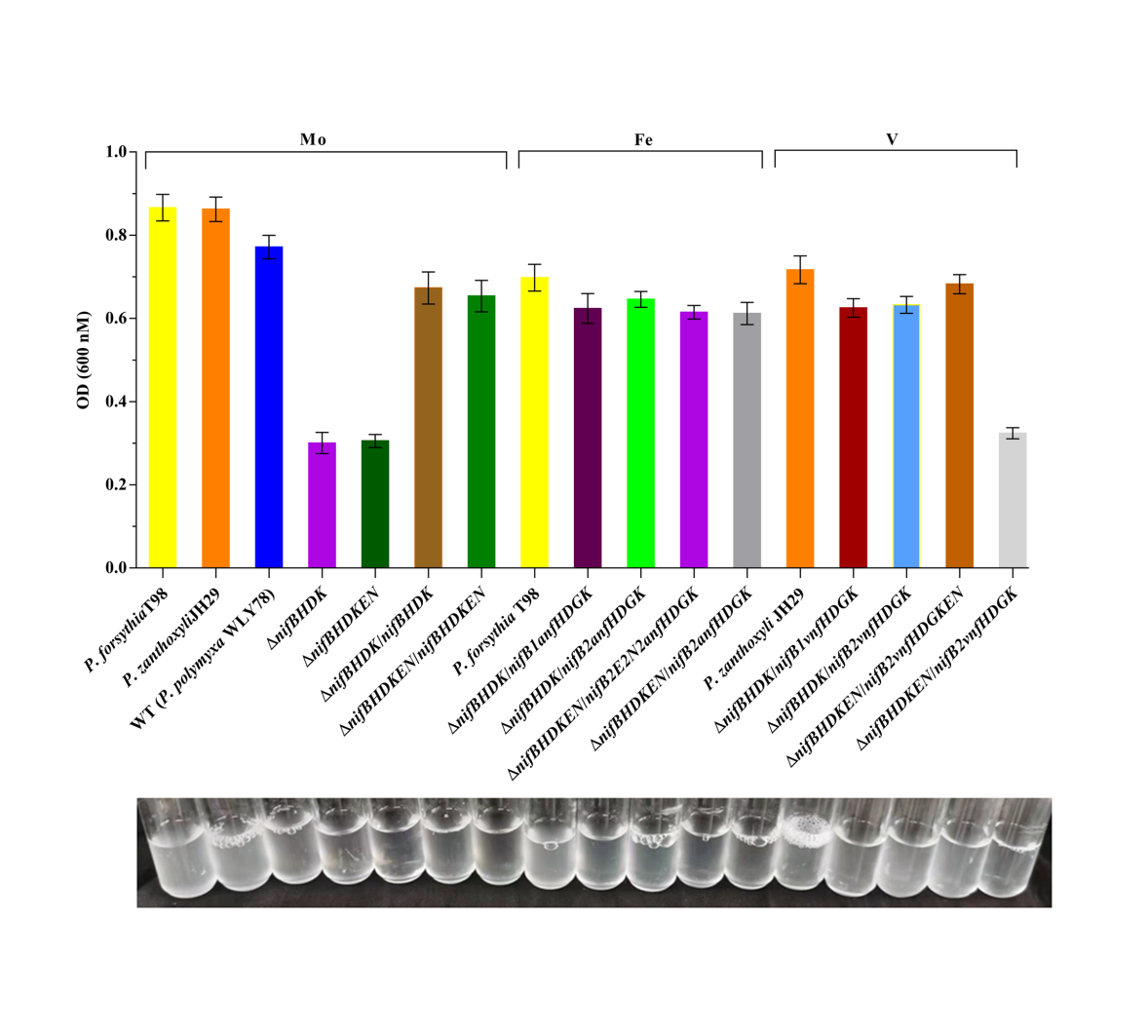


Figure S2. Diazotrophic growth of the Δ*nifBHDK* and Δ*nifBHDKEN* mutants of *P. polymyxa* and the complementary strains carrying *nifB1anfHDGK*, *nifB2anfBHDGK, nifB2E2N2anfBHDGK* from *P. forsythia* T98, and *nifB1vnfHDGK*, *nifB2vnfHDGK,* *nifB2vnfHDGKEN* from *P. zanthoxyli* JH29, respectively.

**a *Paenibacillus zanthoxyli* JH29** ***nifB3* and an additional *nifX*-like gene**

**1 TTGGCGAACGAATCACGCCATCCTTGTTACGACGAGATGGCGCATCATTATTTTGCCCGC**

**61 ATGCATGTGGCGGTAGCGCCGAAATGCAATATCAACTGCAATTATTGCAATATCAAATAT**

**121 GACTGCGTCAGCGAGAGCCGCCCCGGTGTCGTCAGTAAGGTACTTACGCCGCAGGAGGCT**

**181 TACGCCAAGGTGCGTAATACGCTGGCTTCGCTGCCGCAGCTGACCGTGGTCGGCATCGCG**

**241 GGACCCGGCGACCCGCTGGCCAATCCGCAGGAGACATTCGAAACCTTCGGCCTGCTGTCC**

**301 GAAGGCCTGCCCGAGCTGCAGCTGTGCCTCAGTACCAACGGGCTGAAGCTGCCCGATTAT**

**361 TCGGAGGATATCGTCACGCATCGCCCATGTGACCGTCACGGTCAATGCCATCGATCCGCA**

**421 GATCGGGGCGGAGGTATACCGCTCCGTGTTCTTCCGAGGCAAGGCCTACAGGGGCAGGGA**

**481 CGCTGCGGAACTGTTACTGCACAACCAGCTGGAGGGTATCTGGCGGATCGCAGAACGCGG**

**541 CGTAAAGGTCAAAGTCAATTCCGTGCTGATTCCCGGCGTTAATGACAGCCATCTGATCAA**

**601 GGTGACCGAGGCGGTCAAGGCGGCAGGCGCTTTCTCCCATAATATTATGCCGCTGATTCT**

**661 CTCGCCGGGCAGCGTATATGAGAAGGAAGGACGCAAGGAGCCCGCTCCTACGCTGACGCT**

**721 TCAGGTGCTGGCGTACAGCGAGGCGGTTATGCCGGTCATGCGGCATTGCCGCCAATGCCG**

**781 CGCCGACGCGGTCGGGCTGATTGGCGATGATCTTGGCGTCAAGACCGAAAACGCGCCGGC**

**841 CCGCGAATACGGCCCGGCAGCACGCGAGCAGCTGCTTATCCGGCTGGACCGGACGGCCGC**

**901 GGAGAGCAAGCGGCGCGAAGCCAATGCGGGAGAGCAAGGAGAGCGCGGCAGTATACGCAT**

**961 CGCCGTGGCAACGCGCGGCGGGAGCCAGGTGAATGTGCATTTCGGCCATGCCCGCGAGTT**

**1021TCTTGTCTACGAGGTGTCCGGGCAAGAGCAGAAGCTGCTGGGCGTTCGGCGGATTCAGGC**

**1081TTACTGCAACGGCAAGGCCGAATGCGGGGAAGCGGACAAGGAGGCGATTCTGGATGAGAC**

**1141GGTGGCGCTGCTGCAGGATTGCTCCATCGTTCTCTGCGCCGCGATCGGTCCCGGACCGGC**

**1201CGAGCGGCTGGCCAGCGCCGGTCTGACAGCGCTGGCGCGAAAGGGAGAGATCGGCAAGCT**

**1261AATGGAGCAGTGCGCCAAATATCACCGCTTCTTCACCTCCATCTCCTGCTCCCCAAGGCT**

**1321GTCTGCTGATCAGCGGATGGATTTACACGTTCAATGA**

**b *Paenibacillus durus* DSM 1735 *nifB3* gene and an additional *nifX*-like gene**

**1 ATGCATTTCTTGTCTGGCTGCGGCGGACAATCGCCGCCAAGCTCTAACGTACCAGATCGG**

**61 GATATCGTCTTCGCGGTGAAGGACGAGCCTGGACAAATTGATGGAAAGGTGGTATTTGCG**

**121 TTGGCGAACGAATCACGCCATCCTTGTTACGACGAGATGGCGCATCATTATTTTGCCCGC**

**181 ATGCATGTGGCGGTAGCGCCGAAATGCAATATCAACTGCAATTATTGCAATATCAAATAT**

**241 GACTGCGTCAGCGAGAGTCGCCCCGGCGTCGTCAGTAAGGTGCTTACGCCGCAGGAGGCT**

**301 TACGCCAAGGTGCGCAGTACGCTGGCTTCGCTGCCGCAGCTGACTGTGGTCGGCATCGCA**

**361 GGACCCGGCGATCCGCTGGCCAATCCGCGGGAGACGTTCGAAACCTTCGGCCTGCTGTCC**

**421 GAAGGCCTGCCCGATCTGCAGCTGTGCCTCAGTACCAACGGGCTGAAGCTGCCCGATTAT**

**481 TCGGAGGATATCGTCAGGCATCGCATCTCCCATGTGGCCGTCACGGTCAATGCCGTCGAT**

**541 CCGCAGATCGGGGCGGAGGTATACCGCGCTGTGTTCTTCCGAGGTAAGGCCTACAGGGGC**

**601 AGGGAGGCTGCAGAACTGTTACTGCACAACCAGCTGGAGGGCATCCGGCGGATCGCTAGA**

**661 AGTGTGGCGTAAAGGTCAAAGTCAATTCCGTGCTGATTCCCGGCGTTAATGACAGCCATC**

**721 TGATCAAGGTGACCGAGGCGGTCAAGGCGGCAGGCGCTTGCTCCCATAATATTATGCCGC**

**781 TGATTCTCTCGCCTGGCAGCGTATATGAGAAGGAAGGACGCAAGGAGCCCGCTCCTTCGC**

**841 TGACGCTTCAGGTGCAGGCGCGCAGCGAGGCGGTTATGCCGGTCATGCGGCATTGCCGCC**

**901 AATGCCGCGCCGACGCGGTCGGGCTGATTGGCGAAGATCTTGGCGTCAAGACCGGAAACG**

**961 CACCGGCCCGGGAATATGACCCGGCAGCACGCGAGGAGCTGCTTATCCGGCTGGACCGGA**

**1021CGACCGCGGAGCACAAGCGGCGCGAAGCCAATGAGGGAGAGCAAGGAGAGCGCGGCAGCA**

**1081TGCGCATCGCCGTGGCGACGCGCGGCGGGGGCCAGGTGAATGTACATTTCGGACATGCAC**

**1141GCGAGTTTCTTGTCTACGAGGTATCAGGGCAAGAGCAGAAGCTGCTGGGCGTTCGGCGGA**

**1201TTCAGGCTTACTGCAACGGCAAGGCCGAATGCGGGGAAGCGGACAAGGAGGCGATTCTGG**

**1261ATGAGACGGTGGCGCTGCTGCAGGATTGCTCCATCGTTCTCTGCGCCACGATCGGTCCCG**

**1321GACCGGCCGAGCGGCTGGCCCGCGCCGGTCTGACAGCGCTGGCGCGAGAGGGAGAGATCG**

**1381GCAAGCTGCTGGAGCAGTGTGCCAAATATCATCGCTTCTTCAGCTCCATCTCCTGCTCCC**

**1441CACCTTCAGCTTGCCCGTCCGCTGCGCAGCAGCGGTGA**

Figure S3. Nucleotide sequence of DNA fragment containing *nifB3* and an additional *nifX*-like in *P. zanthoxyli* JH29 and *P. durus* DSM 1735. The DNA sequence is presented in the 5'-3' direction. Start and stop codons of translation are highlighted in red and blue, respectively*.* **a** In *P. zanthoxyli* JH29, the *nifB3* encoding a SAM protein is 582 bp (from 1 bp to 582 bp) in length and the *nifX*-like gene encoding a NifX-like protein is 795 bp (from 583 bp to 1357 bp) in length. **b** In *P. durus* DSM 1735, the *nifB3* encoding a SAM protein is 672 bp (from 1 bp to 672 bp) in length and the *nifX*-like gene encoding a NifX-like protein is 789 bp (from 690 bp to 1478 bp) in length.


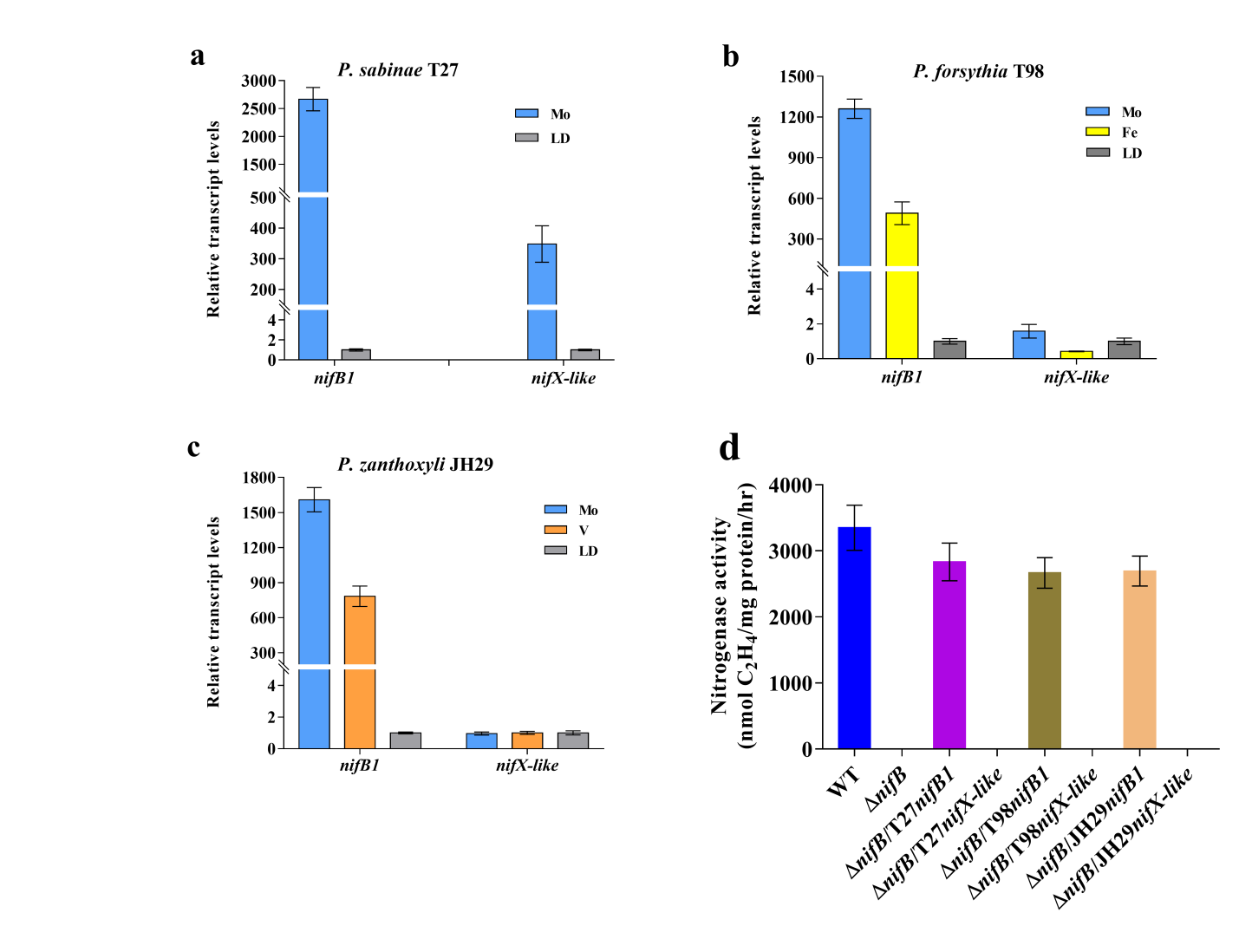


Figure S4. Transcription analysis of the *nifX*-like genes and nitrogenase activities of the *P. polymyxa* Δ*nifB* complementary strains carrying *nifX*-like gene under nitrogen fixation conditions. **a-c** qRT-PCR analysis of the relative transcript levels of the *nifX-*like genes from *P. sabinae* T27, *P. forsythia* T98 and *P. zanthoxyli* JH29 grown in Mo-dependent, Fe-dependent and V-dependent nitrogen fixation conditions, with non-nitrogen fixing conditions of N-rich (LD medium) cultures as negative controls. The data are the mean of three biological replicates. **d** The nitrogenase activity of *P. polymyxa* Δ*nifB* complementary strains carrying *nifX-*like gene from *P. sabinae* T27, *P. forsythia* T98 and *P. zanthoxyli* JH29 was measured by acetylene reduction assay when grown in Mo-dependent medium anaerobically.
